# Using the Hospital Frailty Risk Score to predict length of stay across all adult ages

**DOI:** 10.1371/journal.pone.0317234

**Published:** 2025-01-23

**Authors:** Huda Kutrani, Jim Briggs, David Prytherch, Claire Spice

**Affiliations:** 1 Centre for Healthcare Modelling and Informatics, University of Portsmouth, Portsmouth, United Kingdom; 2 Queen Alexandra Hospital, Portsmouth Hospitals NHS Trust, Portsmouth, United Kingdom; Gabriele d’Annunzio University of Chieti and Pescara: Universita degli Studi Gabriele d’Annunzio Chieti Pescara, ITALY

## Abstract

**Background:**

Hospital Frailty Risk Score (HFRS) has recently been used to predict adverse health outcomes including length of stay (LOS) in hospital. LOS is an important indicator for patient quality of care, the measurement of hospital performance, efficiency and costs. Tools to predict LOS may enable earlier interventions in those identified at higher risk of a long stay. Previous work focused on patients over 75 years of age, but we explore the relationship between HFRS and LOS for all adults.

**Methods:**

This is a retrospective cohort study using data from a large acute hospital during the period from 01/01/2010 to 30/06/2018. The study included patients aged 16 years and older. We calculated HFRS for patients who had been previously admitted to the hospital within the previous 2 years. The study developed Logistic Regression models (crude and adjusted) for nine prediction periods of LOS to assess association between (LOS and HFRS) and (LOS and Charlson Comorbidity Index-CCI), using odds ratios, and AUROC to assess model performance.

**Results:**

An increase in HFRS is associated with prolonged LOS. HFRS alone or combined with CCI were more important predictor of long LOS in most of periods to predict LOS. However, crude HFRS was superior to the models where HFRS was combined with any other variable for LOS in excess of 21 days, which had AUROCs ranging from 0·867 to 0·890. Regarding eight age groups, crude HFRS remained the first or second most effective predictor of long LOS. HFRS alone or combined with CCI was superior to other models for patients older than 44 years for all periods of LOS; whereas for patients younger than 44 years it was superior for all LOS except 45, 60, and 90 days.

**Conclusion:**

This study has demonstrated the utility of HFRS to predict hospital LOS in patients across all ages.

## Introduction

Frailty is a syndrome characterised by reduced strength, physiological function and endurance, which increases an individual’s risk of dependency and death [1]. It is associated with an increased risk of adverse health outcomes for patients in hospital, including longer length of stay (LOS) [1–3].

The UK National Health Service (NHS) estimates that each year 350,000 patients stay in acute hospitals for more than three weeks [4]. In-patients with frailty may have a longer LOS and increased health resource use [5].

Early identification of frailty may prevent, reduce and delay poor health outcomes through early monitoring, investigation and treatment, and aid healthcare organisations in service planning, evaluating health policies, quality improvement and developing markers for epidemiological research [2, 6–8]. Multiple tools to measure frailty have been developed but many require face-to-face assessment which can be difficult in an acute care setting [7–10].

The Hospital Frailty Risk Score (HFRS) is a frailty risk tool calculated using hospital discharge diagnostic codes related to frailty [11]. It was developed based on individuals aged 75 years or older. Subsequently, several other studies have focused on using HFRS with specific conditions across other age ranges, finding that the correlation between HFRS and age was moderate[12], and HFRS remained a significant predictor of longer LOS [10, 12–15]. HFRS has the advantage that it can be calculated in most hospital information systems at low cost, quickly and without additional burden on clinical staff [7, 8, 11, 16, 17].

Previous studies found that using HFRS assisted in improving predictions of poor health outcomes including length of in-hospital stay [6, 7, 9, 10, 17]. Several investigators have used HFRS in combination with common variables such as age, sex and the Charlson Comorbidity Index (CCI) or other comorbidity indices to show that patients with intermediate and high frailty had significantly associated prolonged length of stay compared to patients with low-risk frailty [7, 9, 10, 12, 17, 18]. In the older population the HFRS may outperform comorbidity indices for predicting LOS [18].

Although frailty and comorbidities are associated with poor outcomes and there is overlap between them[18–20] it is sometimes difficult to disentangle frailty and comorbidity [21]. However, frailty tends to increase with age [22–25]. The majority of studies on the use of HFRS have focused on older patients as the original study did, but there is emerging evidence for frailty in younger age groups. Studies recommend screening and understanding of frailty in young and middle-aged adults which may differ from those in older people, and identifying the factors associated with it to prevent frailty progression [19, 20, 24, 26, 27] Increasing frailty in community-dwelling people across almost all age groups has also been described [27]. A quarter of hospitalised younger patients have been found to have frailty and this was associated with an increased length of stay [19].

This study aimed to explore the relationship between Hospital Frailty Risk Score and long length of hospital stay across all adult age groups.

## Materials and methods

### Study design and participants

This was a retrospective cohort study of both elective and non-elective patients who were admitted between 2010 and 2018 to a large acute hospital (Queen Alexandra Hospital in Portsmouth, UK). The study included patients aged 16 years and older who had not registered for the national data opt-out to stop their data being used for research.

**Fig 1:**
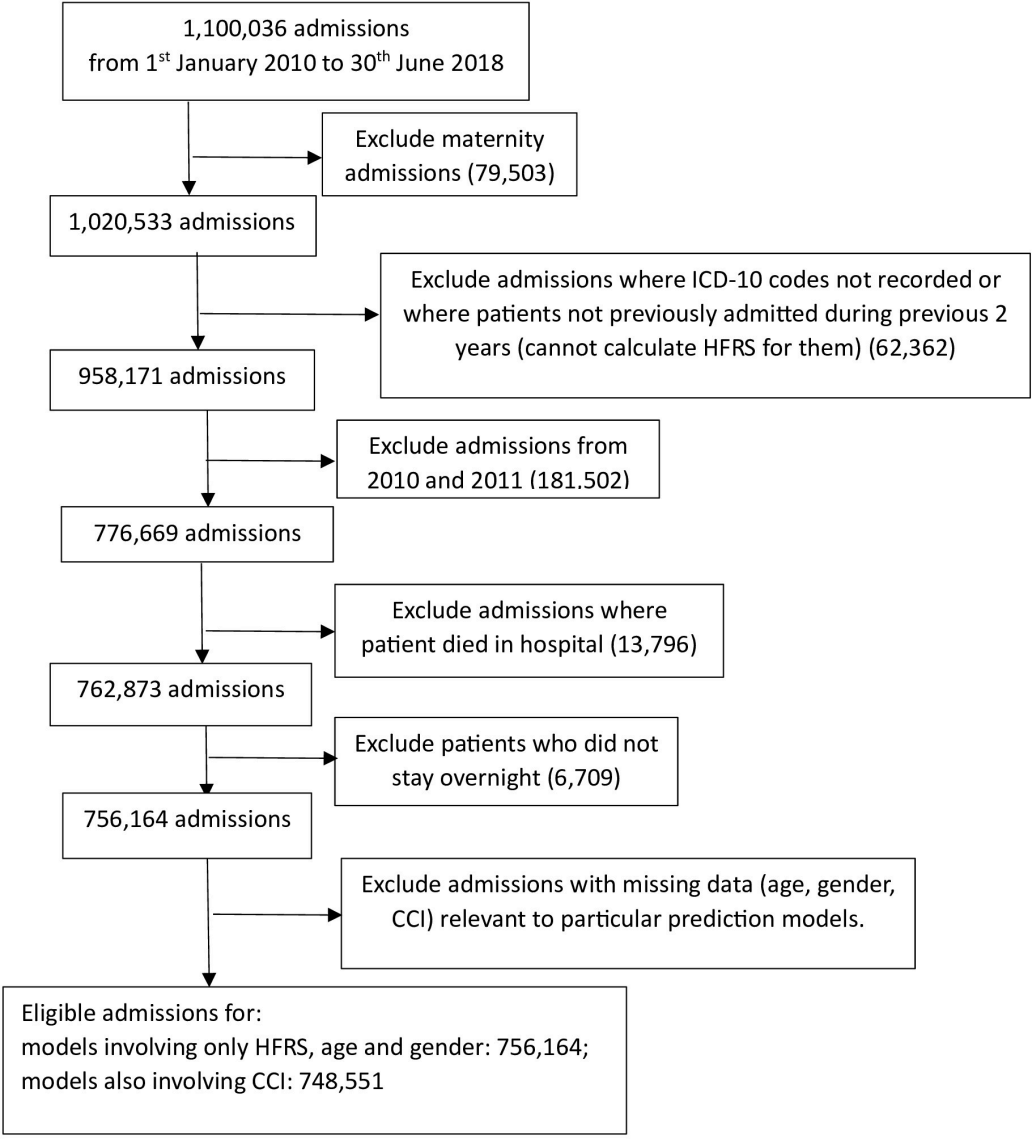
Flowchart of data exclusions.

The full dataset held patient admissions from 1st January 2010 to 30th June 2018 (1,100,036 admissions). Since the calculation of HFRS relies on having 2 previous years’ data for optimal construction of HFRS [17], the analysis was based on patients admitted from 1st January 2012 to 30th June 2018. We excluded patients who died in hospital because they had a shorter length of stay than if they had survived, which may have a negative effect on predicting longer LOS [28]. Other excluded admissions are described in Fig 1.

In addition, we generated 8 age-band sub-datasets (16–24, 25–34, 35–44, and so on up to 85 and over) to evaluate HFRS prediction for the nine periods of LOS.

### HFRS calculation

We used the methodology described by the original study [11]. HFRS was calculated based on ICD-10 diagnoses codes that were documented in patients’ hospital admission records. The final HFRS for each patient is calculated by adding the weighted points together for each code present from the index admission with any diagnosis recorded from previous admissions during the previous two years. HFRS was categorised as low frailty risk (<5), intermediate frailty risk (5–15), and high frailty risk (>15) [11].

### Outcomes and variables

Length of stay (LOS) refers to the total number of days that a patient stays in the hospital per admission [4]. The NHS defines a long stay as LOS > 21 days [4]. Several studies focus on LOS > 10 days [7, 9, 11, 12, 17]. However, here we explore different periods of LOS to provide finer-grained analysis.

The association and predictive ability on LOS of the HFRS tool was evaluated for nine periods of LOS as outcomes of the study. These prediction periods were:

1. LOS >3 days,
2. LOS >7 days,
3. LOS >10 days,
4. LOS >14 days,
5. LOS >21 days,
6. LOS >30 days,
7. LOS >45 days,
8. LOS >60 days and
9. LOS >90 days.

Variables were HFRS, age, gender, and Charlson Comorbidity Index (CCI). CCI is a validated and applicable method of estimating the risk of death from comorbidities of patients based on the ICD-10 diagnosis codes found in administrative data. It includes 17 chronic conditions to predict one-year mortality. The higher the score, the more likely the predicted outcome will result in mortality or higher resource use [29, 30].

### Statistical analysis

Descriptive analysis was used to summarize patients’ characteristics for continuous variables using mean and standard deviation, median and interquartile range (IQR), and frequency and percentage for categorical variables.

Logistic regression models were developed for each of nine LOS periods. Firstly, models related crude HFRS points with each period of LOS. By "crude", we mean not combined with any other data. Secondly, models related HFRS points plus one other variable with each period of LOS. Thirdly, models related crude CCI with each period of LOS. Fourthly, models related CCI plus one other variable with each period of LOS.

Associations between standardized HFRS or standardized CCI and variables with LOS were evaluated by Odds Ratio. Model performance was assessed using AUROC or c-statistic with 95% confidence interval (CI). A value of AUROC below 0.60 indicates no discrimination; values ranging from 0.60–0.69 indicate poor discrimination, values of 0.70–0.79 indicate fair discrimination, values from 0.80–0.89 indicate good discrimination, and values of 0.90 and above indicate excellent discrimination [31]. We compared the AUROC value of the HFRS crude model with other models for each period of LOS. Thus, a higher AUROC indicated better performance of the model.

The strength of the relationship between HFRS, CCI, and age for all patients and each age group was assessed using the Pearson Correlation Coefficient. A value below 0.29 indicates weak correlation; values ranging from 0.3–0.49 indicate moderate correlation, and values of 0.5 and above indicate strong correlation. Data manipulation and logistic regression modelling were performed using RStudio version 4.2.1.

### Ethics

The study included patients aged 16 years and older who had not registered for the national data opt-out to stop their data being used for research. We accessed to data from the Portsmouth CORE-D routine care data repository on 1st November 2022. Also, we did not know information that could identify individual patients during or after data collection.

In addition, the dataset used in this study was covered by existing ethical approval granted by an NHS Research Ethics Committee in April 2021. The research database used is the Portsmouth CORE-D routine care data repository. REC reference is 21/SC/0080. IRAS project ID is 281193. The data is available from Portsmouth Hospital University NHS Trust under a data sharing agreement.

## Results

Data included patients aged 16 years and older who were admitted to Queen Alexandra Hospital in Portsmouth, UK. Patients’ characteristics in the dataset and each of the length of stay period subsets are presented in Table 1. Mean Hospital Frailty Risk Scores (HFRS) increased with longer LOS. In general, CCI increased with LOS but then decreased (after LOS > 21 days). The number of patients in each LOS group obviously diminished as LOS got longer since some patients were discharged before they reached that LOS threshold.

**Table 1:** Characteristics of patients by prediction length of stay periods.

	All patients n = 756,164	Length of Stay (LOS) group								
		LOS >3days n = 119,460	LOS >7days n = 65,971	LOS >10days n = 48,255	LOS >14days n = 34,258	LOS >21days n = 20,743	LOS >30days n = 12,472	LOS >45days n = 6,028	LOS >60days n = 3,137	LOS >90days n = 957
**HFRS**										
Mean (SD)	3.5 (6.2)	8.9 (9.2)	11.1 (9.7)	11.9 (9.7)	12.7 (9.8)	13.7 (9.9)	14.4 (10.0)	14.9 (10.1)	15.0 (10.0)	15.2 (9.5)
Median (IQR)	0.80 (0.0–4.0)	6.0 (1.8–13.3)	8.6(3.4–16.3)	9.6(4.2–17.4)	10.6(5.0–18.3)	11.8(6.0–19.4)	12.6(6.6–20.2)	13.4(7.1–20.7)	13.4(7.5–20.5)	13.7(8.1–20.6)
**Age in years**										
Mean (SD)	62.3 (19.0)	71.1 (19.4)	74.6 (15.9)	75.8 (15.4)	76.6 (14.9)	77.1 (14.7)	76.8 (14.9)	76.4 (14.8)	75.2 (15.0)	72.9 (15.7)
Median (IQR)	66.0(50.0–77.0)	75.0 (61.0–84.0)	79.0(66.0–86.0)	80.0(68.0–87.0)	80.0(69.0–87.0)	81.0(70.0–88.0)	81.0(70.0–87.0)	80.0(69.0–87.0)	79.0(67.0–86.0)	77.0(64.0–85.0)
**CCI**										
Mean (SD)	2.3 (6.1)	5.6 (9.3)	6.5 (10.1)	6.8 (10.3)	6.8 (10.3)	6.8 (10.3)	6.4 (10.0)	5.9 (9.6)	5.5 (9.2)	4.8 (8.8)
Median (IQR)	0.0(0.0–0.0)	0.0 (0.0–9.0)	0.0(0.0–11.0)	0.0(0.0–12.0)	0.0(0.0–12.0)	0.0(0.0–13.0)	0.0(0.0–11.0)	0.0(0.0–11.0)	0.0(0.0–10.0)	0.0(0.0–7.0)
**Gender**										
Female No (%)	411792(54.5%)	63524 (53.2%)	35426 (53.7%)	25996 (53.9%)	18448 (53.9%)	11029 (53.2%)	6533(52.4%)	3025 (50.2%)	1550 (49.4%)	443(46.3%)
Male No (%)	344372(45.5%)	55936 (46.8%)	30545 (46.3%)	22256 (46.1%)	15810 (46.1%)	9714 (46.8%)	5939 (47.6%)	3003 (49.8%)	1587 (50.6%)	514 (53.7%)

**HFRS:** Hospital frailty risk score; **CCI:** Charlson Comorbidity Index

**Fig 2:**
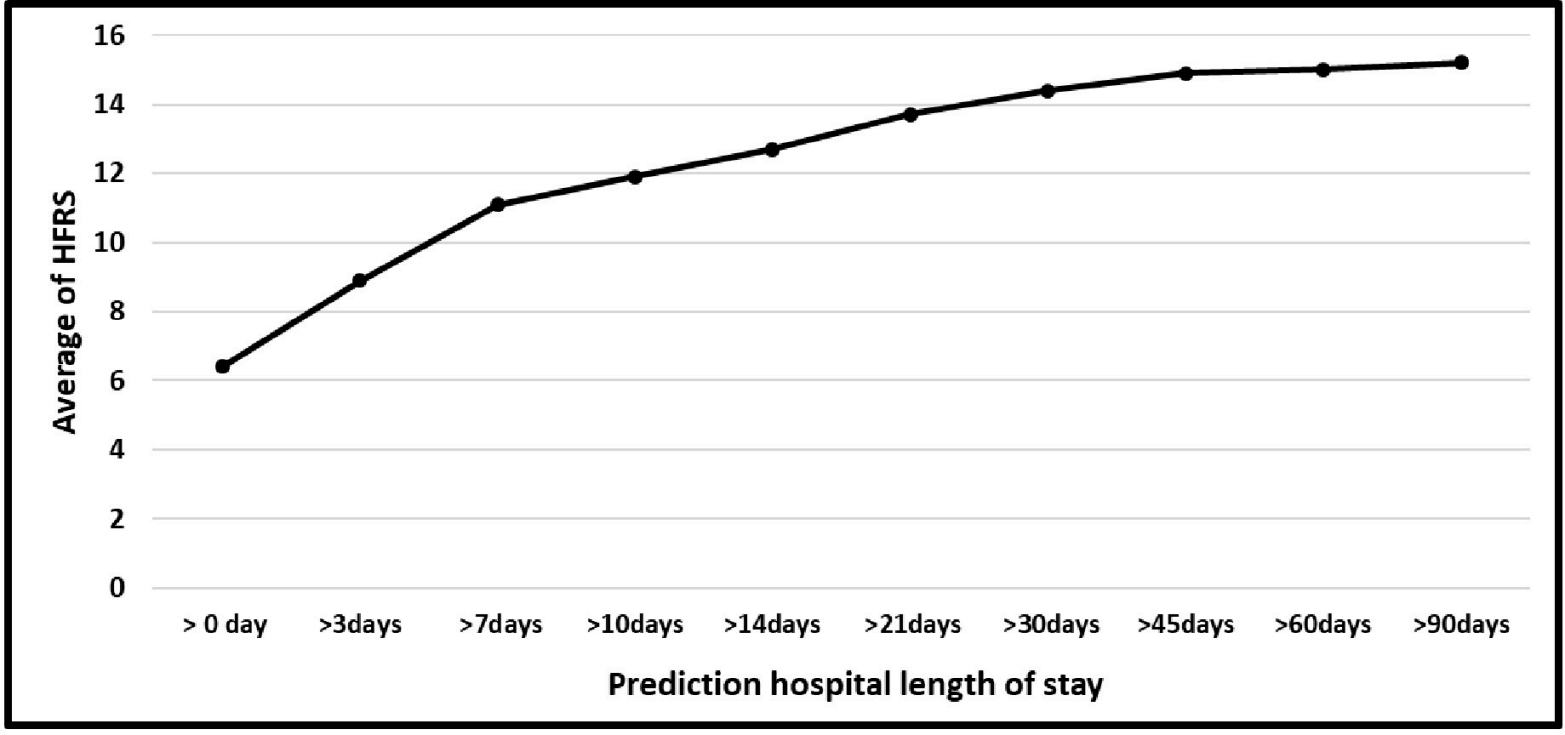
Relation between Hospital Frailty Risk Scores and nine prediction periods of LOS.

The relationship between HFRS and nine periods of LOS is presented in Fig 2. We noted that an increase in HFRS is associated with longer LOS in the hospital. All nine periods of LOS had an average HFRS in the "intermediate risk" band.

Table 2 Demonstrates the risk of frailty increased with increasing age, as would be expected, but intermediate and high risk of frailty were identified in all age groups.

**Table 2:** Patients with risk of frailty according to age groups.

Subset data	No. of admissions	Proportion of No. of admissions	Number of unique patients	Number of admissions with HFRS greater than 0	Hospital Frailty Risk Score category		
				No. (%)	Low risk (<5)	Intermediate risk (5–15)	High risk (>15)
all ages	756,164	100%	220,408	433,309 (57.3%)	604,602 (80.0%)	109,244 (14.4%)	42,318 (5.6%)
16–24 years	34,766	4.6%	17,589	17,209 (49.5%)	32,129 (92.4%)	2,363 (6.8%)	274 (0.8%)
25–34 years	57,740	7.6%	26,531	29,223 (50.6%)	52,699 (91.3%)	4,581 (7.9%)	460 (0.8%)
35–44 years	61,390	8.1%	25,930	31,487 (51.3%)	55,185 (89.9%)	5,534 (9.0%)	671 (1.1%)
45–54 years	97,330	12.9%	35,572	52,171 (53.6%)	85,584 (87.9%)	9,864 (10.1%)	1,882(1.9%)
55–64 years	124,334	16.4%	41,737	68,756 (55.3%)	107,953 (86.8%)	13,314 (10.7%)	3,067 (2.5%)
65–74 years	160,610	21.2%	46,771	90,102 (56.1%)	131,948 (82.2%)	22,529 (14.0%)	6,133 (3.8%)
75–84 years	140,460	18.6%	40,913	90,744 (64.6%)	99,865 (71.1%)	27,860 (19.8%)	12,735 (9.1%)
≥ 85 years	79,534	10.5%	25,110	61,991 (77.9%)	39,239 (49.3%)	23,199 (29.2%)	17,096 (21.5%)

**Fig 3:**
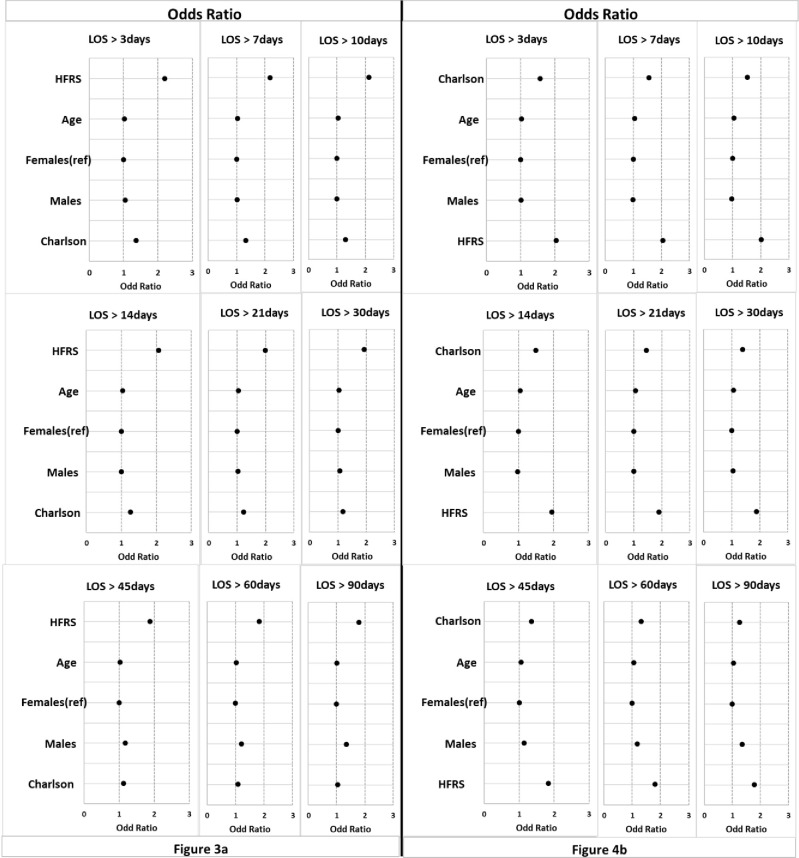
Odds ratio for nine prediction periods of LOS. (a) is HFRS (b) is CCI.

Fig 3A compares crude HFRS with HFRS combined with one other variable. HFRS alone was the first or second most effective predictor of long LOS for all periods with odds ratios between 1.8–2.2. CCI was the second most effective predictor of length of stay (odds ratio ranged from 1.18–1.37). The results show that age is not an important additional predictor compared to other variables for any period.

Fig 3B compares crude CCI with CCI combined with one other variable. Combining CCI with gender was the most effective predictor of long LOS in excess of 45 days. Combining CCI with HFRS was the first or second most effective predictor for all periods of LOS (odds ratios range from 1.79–2.05). The results again show that age is not an important additional predictor compared to other variables for any period. CCI alone was the second most effective predictor (odds ratios ranged from 1.27 to 1.58).

**Fig 4:**
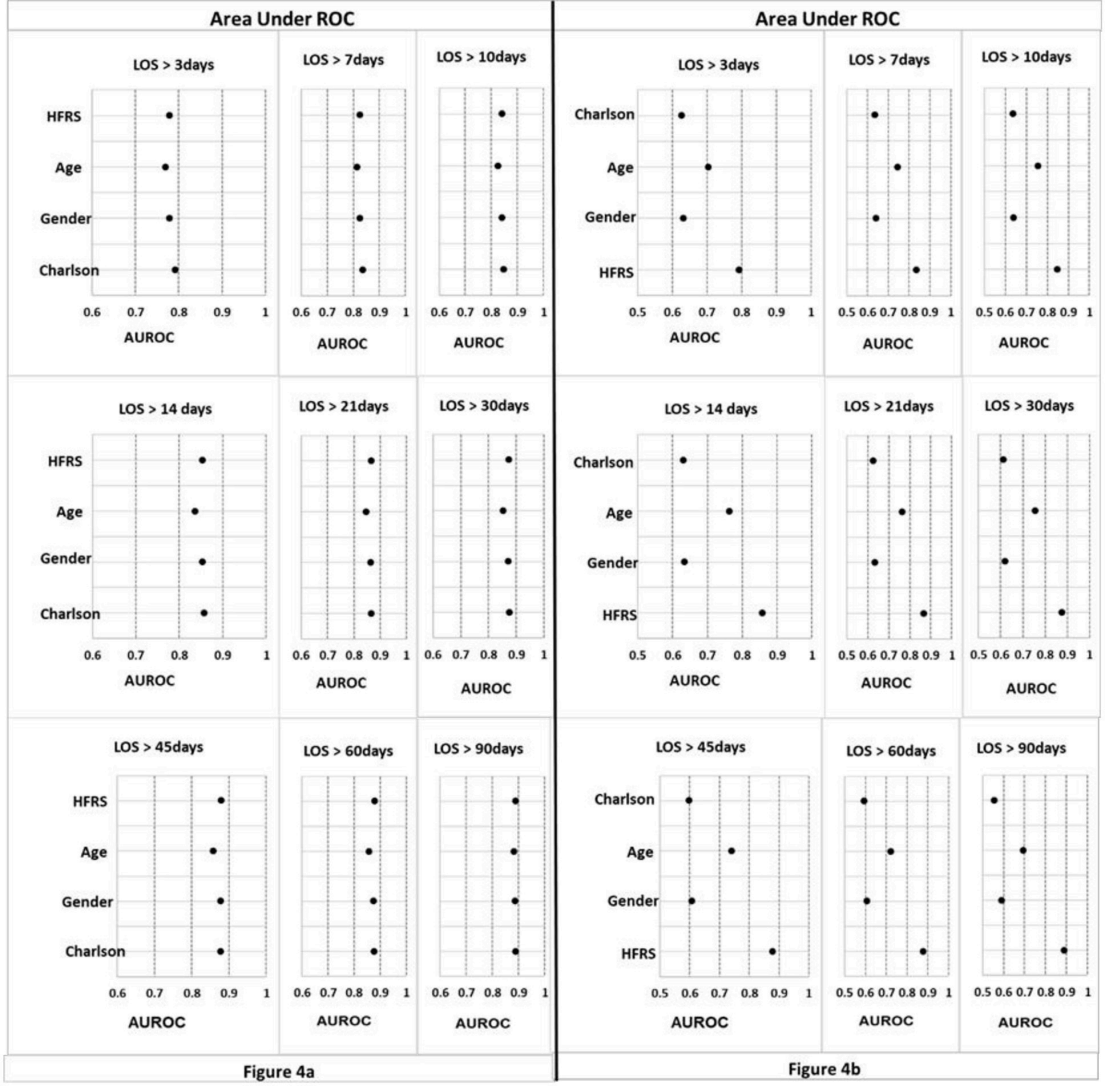
Area Under ROC for nine periods of prediction long length of stay.

The Area Under ROC curve (AUROC) was used to evaluate the performance of the models. Fig 4A and S1 Table summarise the evaluation of the predictive power of HFRS alone and with other variables. In general, the predictive power of crude HFRS increased with LOS: AUROC = 0.779 for LOS>3 days, rising to 0.890 for LOS>90 days. Crude HFRS was superior to the models where HFRS was combined with any other variable for LOS in excess of 21 days. However, the AUROC value of HFRS combined with CCI model was superior to the crude HFRS and models where HFRS was combined with any other variable for LOS less than 21days.

Fig 4B and S1 Table summarise the evaluation of the predictive power of CCI alone and with other variables. In general, the predictive power of CCI combined with HFRS increased with LOS. In each LOS period, the AUROC value of the combined CCI and HFRS model was superior to the crude CCI or models where CCI was combined with any other variable. In addition, all AUROCs were statistically significant which indicate reliable predictive performance and improved AUROC. This result shows that HFRS significantly improves models’ performance to predict LOS.

Regression model results for each period of length of stay and across the eight age groups are summarised in S2 and S3 Tables and S1 Fig. Overall, crude HFRS was a more effective predictor of long length of stay for all periods of LOS and all age groups than HFRS combined with age or CCI.

S3 Table and S1 Fig summarized regression model results for crude CCI, or CCI combined with one other variable. Combining CCI with gender was the most effective predictor of long LOS for patients younger than 65 years for all periods of LOS—the odds ratios ranged from 1.18 to 3.36. Combining CCI with HFRS was the first or second most effective predictor for all periods of LOS for all age groups (odds ratios ranged from 1.46 to 3.09).

S4 and S5 Tables and S2 Fig summarize the evaluation of the predictive power of crude HFRS or crude CCI and when combined with other variables for eight age groups. HFRS alone had discrimination (at least fair, often good, and sometimes excellent) for all periods of LOS and for all age groups. Also, crude HFRS, or HFRS combined with CCI, was superior to the models where HFRS was combined with any other variable: (i) for patients older than 44 years for all periods of LOS and (ii) for patients younger than 44 years for LOSs less than 60 days—AUROCs ranging from 0.700 to 0.923.

Moreover, in each LOS period and for all age groups, the AUROC of the model combining CCI with HFRS model was superior to the crude CCI model, or models where CCI was combined with any other variable. This result shows that HFRS significantly improves models’ performance to predict LOS for all age groups.

Additional analyses were performed based on elective admissions, non-elective admissions, and index admission. We obtained the same study results, crude HFRS or HFRS combined with CCI remained a significant predictor of longer LOS for elective admission, non-elective admissions, and index admission; as shown in S6–S13 Tables.

The correlation between HFRS, CCI, and age is summarised in S14 Table. In general, the Pearson’s correlation for continuous HFRS and CCI was weak in all age groups. However, the correlation between (HFRS and age) and (CCI and age) was also weak.

## Discussion

The Hospital Frailty Risk Score (HFRS) is associated with adverse outcomes for older people [5, 7, 9, 11, 17] including longer length of stay. Whilst less common, frailty has been described in younger people in hospital [19, 20, 24, 26, 27] and is associated with increased healthcare resource use [5, 7]. This study explores the relationship between HFRS and long LOS across all adult age groups and found that an increase in HFRS is associated with longer LOS in the hospital—as shown in Table 1 and Fig 2. The proportions in our study of patients with an intermediate or high-risk score for frailty are in keeping with those described by others using clinically applied frailty tools for people in hospital in younger groups [25, 32].

Most studies used HFRS to predict prolonged length of stay with common variables such as age, sex, and the Charlson Comorbidity Index (CCI) [6–11, 17]. Our study aimed to evaluate the predictive power of HFRS to predict longer LOS by comparing the AUROC results obtained from a crude HFRS model to other models for each period of LOS. We found that HFRS alone (or when combined with CCI) was a better predictor of long length of stay than the other variables for all periods of length of stay. HFRS alone (or combined with CCI) had the highest discrimination (fair and good) in all periods compared to other variables. We can conclude that HFRS is a significant predictor of long length of stay in hospital for all nine periods of LOS considered and across all ages. This is in keeping with other studies that used HFRS across all ages that focused on patients with specific conditions [10, 12–14, 33]. In general surgical in-patients, the HFRS remained predictive of outcomes, including LOS, even when CCI was adjusted for [10]. This may be because, whilst the HFRS has some cross over with CCI variables such as dementia, it extends the identification of frailty with the broader spectrum of associated coded conditions. The findings are not universal and, in a community based younger population, the HFRS did not fully capture frailty when compared to a frailty index [15]. The HFRS is a good and reliable proxy measure of patients at risk of high resource use in hospital and our study demonstrates it can be useful across all adult age groups. The association of the HFRS with long LOS is not surprising as it was developed based on older patients with high resource use. We know that frailty is strongly associated with comorbidity [13, 29]. We have shown that HFRS combined with CCI has the highest discrimination for patients older than 25 years for all periods of LOS considered. This agrees with studies that used HFRS and found that HFRS and CCI remained a significant predictor of long LOS [10, 18].

Overall, models containing HFRS alone or combined with one other variable were superior to models with CCI alone or combined with age or gender across all LOS and for all age groups. HFRS was superior to other variables across all lengths of stay for groups 44+ years and for most lengths of stay (but not the very long LOS) in younger cohorts. These results show that HFRS significantly improves models’ performance to predict LOS across eight of the nine age groups.

Our study explored if the HFRS alone, or HFRS combined with CCI models, delivered the same study results for a non-elective admissions dataset, an elective admissions dataset, and an index admission dataset. We found that it does not matter whether we include all admissions or just non-elective admissions or index admissions, the results are still same. Also, all admissions delivered the best results in terms of AUROC for most LOS periods across all ages and in 8 age groups.

Furthermore, we found the correlation between HFRS and CCI was weak in all patients and in eight age groups. However, HFRS alone or combined with CCI were shown in our study to be able to predict long LOS across all ages, because both HFRS and CCI are associated with poor outcomes.

Strengths of this study include exploring the use of the HFRS across all age groups, since few studies have applied the HFRS in populations younger than 65 years. An optimal construction of the score was used with administrative data from previous hospitalisations over 2 years [17]. Previous studies have used a cut off of 10 or more days to indicate prolonged LOS [6, 7, 9, 11, 17] which may not be clinically relevant enough to capture very long hospital stays associated with patient complexity. We have used nine different thresholds of LOS and have demonstrated that HFRS is predictive of prolonged LOS, with particularly good performance for predicting LOS in excess of 21 days.

While this study demonstrated a higher HFRS is associated with increasing LOS across most adult age groups it does have some limitations. Those who died were excluded to avoid reducing utility to detect association of HFRS with longer LOS. This may reduce routine service or clinical utility. Further research focused on all patients would clarify whether this association persists. This study uses a large amount of data but it is restricted to one hospital, so further research is warranted to see whether the results are replicated in different settings.

There are already several routinely used electronic[23] and clinical tools to identify cohorts of older patients with frailty or individuals with frailty [34]. The identification of younger hospital in-patients with frailty at increased risk of length of stay may be helpful for focusing future interventions to reduce LOS, enhance clinical management and highlight specific populations where service developments could be centred.

## Conclusion

Even though the HFRS was developed and constructed for older people (based on a cluster analysis of patients with high resource use in hospitals [11, 17], our findings showed that the HFRS is a good measure of patients at risk of high resource use in hospitals across all ages and is associated with long LOS. Although risk factors or characteristics of frailty in younger populations may differ from those in older adults [20, 21, 25, 27, 28], this study has demonstrated the utility of the Hospital Frailty Risk Score in predicting long length of stay across all ages of patients in an in-patient hospital setting. Further research is required to understand whether this is of clinical or service development benefit.

## Supporting information

S1 Table Area Under ROC with 95% CI for 9 periods of prediction long length of stay across all ages. (a) for HFRS alone or combined with one other variable (b) for CCI alone or combined with one other variable.
(DOCX)

S2 Table (S2a-S2d Tables). Logistics regression results (odds ratio) from models HFRS alone or combined with one other variable (age, gender, CCI) for each period of LOS and each age groups.
(DOCX)

S3 Table (S3a-S3d Tables). Logistics regression results (odds ratio) from models CCI alone or combined with one other variable (age, gender, HFRS) for each period of LOS and each age groups.
(DOCX)

S4 Table (S4a-S4d Tables). Area Under ROC for 9 periods of long length of stay and 8 age groups for models HFRS alone or combined with one other variable (age, gender, CCI).
(DOCX)

S5 Table (S5a-S5d Tables). Area Under ROC for 9 periods of long length of stay and 8 age groups for models CCI alone or combined with one other variable (age, gender, HFRS).
(DOCX)

S6 Table (S6a-S6f Tables). Area Under ROC for 9 periods of long length of stay for models HFRS alone or combined with one other variable and models CCI alone or combined with one other variable for all admissions, non-elective admissions, and elective admissions.
(DOCX)

S7 Table (S7a-S7d Tables). Area Under ROC for 9 periods of long length of stay and 8 age groups for models HFRS alone or combined with one other variable (age, gender, CCI) for non-elective admissions.
(DOCX)

S8 Table (S8a-S8d Tables). Area Under ROC for 9 periods of long length of stay and 8 age groups for models CCI alone or combined with one other variable (age, gender, HFRS) for non-elective admissions.
(DOCX)

S9 Table (S9a-S9d Tables). Area Under ROC for 9 periods of long length of stay and 8 age groups for models HFRS alone or combined with one other variable (age, gender, CCI) for elective admissions.
(DOCX)

S10 Table (S10a-S10d Tables). Area Under ROC for 9 periods of long length of stay and 8 age groups for models CCI alone or combined with one other variable (age, gender, HFRS) for elective admissions.
(DOCX)

S11 Table (S11a-S11d Tables). Area Under ROC for 9 periods of long length of stay for models HFRS alone or combined with one other variable and models CCI alone or combined with one other variable for all admissions, and index admission.
(DOCX)

S12 Table (S12a-S12d Tables). Area Under ROC for 9 periods of long length of stay and 8 age groups for models HFRS alone or combined with one other variable (age, gender, CCI) for index admission.
(DOCX)

S13 Table (S13a-S13d Tables). Area Under ROC for 9 periods of long length of stay and 8 age groups for models CCI alone or combined with one other variable (age, gender, HFRS) for index admission.
(DOCX)

S14 Table Correlation between HFRS, CCI, and age. (DOCX)

S1 Fig Logistics regression results (odds ratio) for nine prediction periods of LOS and eight age groups. (a) is HFRS models (b) is CCI models.
(DOCX)

S2 Fig Area Under ROC for 9 periods of long length of stay and 8 age groups (a) is HFRS models (b) is CCI models.
(DOCX)
