# Supplementary material for: Using the Hospital Frailty Risk Score to predict length of stay across all adult ages
Source: PLoS One. 2025 Jan 23;20(1):e0317234. doi: 10.1371/journal.pone.0317234 (PMC11756769; doi:10.1371/journal.pone.0317234)
Supplement: S1 Table — (a) for HFRS alone or combined with one other variable (b) for CCI alone or combined with one other variable. (DOCX) [file pone.0317234.s001.docx]

**S1 Table: (S1a & S1b) Tables. Area Under ROC with 95% CI for 9 periods of prediction long length of stay across all ages. (a) for HFRS alone or combined with one other variable (b) for CCI alone or combined with one other variable.**

S1a Table. Area Under ROC with 95% CI for 9 periods of prediction long length of stay for HFRS alone or combined with one other variable (age, gender, CCI) across all ages.

| **Outcomes** | **Length of Stay (LOS) group** | | | |
| --- | --- | --- | --- | --- |
|  | **HFRS alone** | **HFRS+Age** | **HFRS+Gender** | **HFRS+CCI** |
|  | **AUROC (95%CI)** | **AUROC (95%CI)** | **AUROC (95%CI)** | **AUROC (95%CI)** |
| **LOS>3 days** | 0.779 | 0.769 | 0.779 | 0.792 |
|  | (0.773-0.780) | (0.768-0.771) | (0.777-0.780) | (0.790-0.795) |
| **LOS>7 days** | 0.827 | 0.813 | 0.827 | 0.835 |
|  | (0.826-0.829) | (0.811-0.815) | (0.825-0.830) | (0.833-0.837) |
| **LOS>10 days** | 0.841 | 0.826 | 0.841 | 0.847 |
|  | (0.839-0.843) | (0.825-0.833) | (0.840-0.847) | (0.845-0.851) |
| **LOS>14 days** | 0.853 | 0.836 | 0.853 | 0.857 |
|  | (0.851-0.855) | (0.835-0.838) | (0.851-0.855) | (0.855-0.860) |
| **LOS>21 days** | 0.867 | 0.846 | 0.865 | 0.865 |
|  | (0.865-0.869) | (0.844-0.848) | (0.863-0.866) | (0.863-0.868) |
| **LOS>30 days** | 0.875 | 0.853 | 0.871 | 0.874 |
|  | (0.873-0.877) | (0.851-0.855) | (0.870-0.873) | (0.872-0.877) |
| **LOS>45 days** | 0.880 | 0.857 | 0.878 | 0.877 |
|  | (0.878-0.883) | (0.855-0.859) | (0.877-0.879) | (0.873-0.881) |
| **LOS>60 days** | 0.880 | 0.856 | 0.874 | 0.877 |
|  | (0.878-0.883) | (0.855-0.858) | (0.873-0.875) | (0.872-0.882) |
| **LOS>90 days** | 0.890 | 0.882 | 0.888 | 0.889 |
|  | (0.888-0.893) | (0.880-0.885) | (0.886-0.900) | (0.881-0.907) |

**HFRS:** Hospital frailty risk score; **CCI:** Charlson Comorbidity Index

S1b Table. Area Under ROC for 9 periods of prediction long length of stay for CCI alone or combined with one other variable (age, gender, HFRS) across all ages.

| **Outcomes** | **Length of Stay (LOS) group** | | | |
| --- | --- | --- | --- | --- |
|  | **CCI alone** | **CCI+Age** | **CCI+Gender** | **CCI+HFRS** |
|  | **AUROC (95%CI)** | **AUROC (95%CI)** | **AUROC (95%CI)** | **AUROC (95%CI)** |
| **LOS>3 days** | 0.628 | 0.703 | 0.632 | 0.792 |
|  | (0.626-0.629) | (0.700-0.704) | (0.631-0.635) | (0.790-0.795) |
| **LOS>7 days** | 0.637 | 0.743 | 0.641 | 0.835 |
|  | (0.635-0.639) | (0.741-0.745) | (0.639-0.644) | (0.833-0.837) |
| **LOS>10 days** | 0.636 | 0.756 | 0.640 | 0.847 |
|  | (0.633-0.638) | (0.754-0.759) | (0.638-0.643) | (0.845-0.851) |
| **LOS>14 days** | 0.631 | 0.762 | 0.635 | 0.857 |
|  | (0.629-0.633) | (0.758-0.764) | (0.633-0.637) | (0.855-0.860) |
| **LOS>21 days** | 0.626 | 0.762 | 0.633 | 0.865 |
|  | (0.622-0.630) | (0.759-0.765) | (0.630-0.636) | (0.863-0.868) |
| **LOS>30 days** | 0.613 | 0.755 | 0.620 | 0.874 |
|  | (0.609-0.617) | (0.751-0.759) | (0.618-0.623) | (0.872-0.877) |
| **LOS>45 days** | 0.598 | 0.741 | 0.607 | 0.877 |
|  | (0.591-0.605) | (0.736-0.744) | (0.598-0.615) | (0.873-0.881) |
| **LOS>60 days** | 0.593 | 0.720 | 0.608 | 0.877 |
|  | (0.587-0.602) | (0.712-0.725) | (0.594-0.614) | (0.872-0.882) |
| **LOS>90 days** | 0.556 | 0.693 | 0.593 | 0.889 |
|  | (0.552-0.568) | (0.681-0.709) | (0.581-0.612) | (0.881-0.907) |

**HFRS:** Hospital frailty risk score; **CCI:** Charlson Comorbidity Index
