# Supplementary material for: Using the Hospital Frailty Risk Score to predict length of stay across all adult ages
Source: PLoS One. 2025 Jan 23;20(1):e0317234. doi: 10.1371/journal.pone.0317234 (PMC11756769; doi:10.1371/journal.pone.0317234)
Supplement: S2 Table — Logistics regression results (odds ratio) from models HFRS alone or combined with one other variable (age, gender, CCI) for each period of LOS and each age groups. (DOCX) [file pone.0317234.s002.docx]

**S2 Table: (S2a-S2d) Tables. Logistics regression results (odds ratio) from models HFRS alone or combined with one other variable (age, gender, CCI) for each period of LOS for each age groups.**

S2a Table. Logistics regression results (odds ratio) for 9 prediction periods of LOS and 8 age groups for HFRS alone

| **Subset data** | **HFRS alone models** | | | | | | | | |
| --- | --- | --- | --- | --- | --- | --- | --- | --- | --- |
|  | **Length of Stay (LOS) group** | | | | | | | | |
|  | **LOS >3 days** | **LOS >7 days** | **LOS >10 days** | **LOS >14 days** | **LOS >21 days** | **LOS >30 days** | **LOS >45 days** | **LOS >60 days** | **LOS >90 days** |
| All ages | 2.20 | 2.19 | 2.13 | 2.07 | 2.00 | 1.94 | 1.88 | 1.84 | 1.80 |
| 16-24 years | 2.14 | 2.19 | 2.21 | 2.29 | 2.29 | 2.15 | 2.21 | 2.28 | 2.16 |
| 25-34 years | 2.59 | 2.75 | 2.77 | 2.93 | 2.98 | 3.04 | 3.18 | 2.85 | 2.88 |
| 35-44 years | 2.5 | 2.51 | 2.43 | 2.53 | 2.58 | 2.65 | 2.63 | 2.68 | 3.08 |
| 45-54 years | 2.33 | 2.39 | 2.35 | 2.35 | 2.31 | 2.27 | 2.29 | 2.27 | 2.26 |
| 55-64 years | 2.32 | 2.30 | 2.26 | 2.22 | 2.18 | 2.17 | 2.16 | 2.14 | 2.20 |
| 65-74 years | 2.17 | 2.18 | 2.15 | 2.11 | 2.08 | 2.04 | 2.02 | 2.00 | 1.93 |
| 75-84 years | 1.90 | 1.89 | 1.85 | 1.81 | 1.78 | 1.75 | 1.70 | 1.68 | 1.67 |
| ≥85years | 1.70 | 1.62 | 1.58 | 1.54 | 1.51 | 1.49 | 1.48 | 1.47 | 1.49 |

**HFRS:** Hospital frailty risk score; **CCI:** Charlson Comorbidity Index

S2b Table. Logistics regression results (odds ratio) for 9 prediction periods of LOS and 8 age groups for age when combined with HFRS

| Subset data | **age (from HFRS+age models)** | | | | | | | | |
| --- | --- | --- | --- | --- | --- | --- | --- | --- | --- |
|  | **Length of Stay (LOS) group** | | | | | | | | |
|  | **LOS >3 days** | **LOS >7 days** | **LOS >10 days** | **LOS >14 days** | **LOS >21 days** | **LOS >30 days** | **LOS >45 days** | **LOS >60 days** | **LOS >90 days** |
| All ages | 1.02 | 1.03 | 1.04 | 1.04 | 1.04 | 1.04 | 1.03 | 1.02 | 1.01 |
| 16-24 years | 1.00 | 0.99 | 0.98 | 0.97 | 1.00 | 1.05 | 1.05 | 1.14 | 1.17 |
| 25-34 years | 1.01 | 1.02 | 1.01 | 1.02 | 1.03 | 1.01 | 1.02 | 0.98 | 0.88 |
| 35-44 years | 1.02 | 1.02 | 1.01 | 1.01 | 1.01 | 1.00 | 1.02 | 0.97 | 0.88 |
| 45-54 years | 1.01 | 1.02 | 1.02 | 1.03 | 1.02 | 1.02 | 1.05 | 1.03 | 1.04 |
| 55-64 years | 1.04 | 1.04 | 1.04 | 1.04 | 1.03 | 1.01 | 1.01 | 1.01 | 1.01 |
| 65-74 years | 1.01 | 1.02 | 1.03 | 1.03 | 1.03 | 1.04 | 1.02 | 1.00 | 1.00 |
| 75-84 years | 1.04 | 1.05 | 1.05 | 1.05 | 1.05 | 1.05 | 1.05 | 1.04 | 1.05 |
| ≥85years | 1.04 | 1.04 | 1.04 | 1.04 | 1.04 | 1.03 | 1.01 | 0.99 | 1.00 |

**HFRS:** Hospital frailty risk score; **CCI:** Charlson Comorbidity Index

S2c Table. Logistics regression results (odds ratio) for 9 prediction periods of LOS and 8 age groups for gender when combined with HFRS

| Subset data | **Gender (from HFRS + gender models) Females Ref = 1** | | | | | | | | |
| --- | --- | --- | --- | --- | --- | --- | --- | --- | --- |
|  | **Length of Stay (LOS) group** | | | | | | | | |
|  | **LOS >3 days** | **LOS >7 days** | **LOS >10 days** | **LOS >14 days** | **LOS >21 days** | **LOS >30 days** | **LOS >45 days** | **LOS >60 days** | **LOS >90 days** |
| All ages | 1.05 | 1.02 | 1.01 | 1.01 | 1.04 | 1.08 | 1.18 | 1.21 | 1.36 |
| 16-24 years | 1.37 | 1.74 | 1.92 | 2.17 | 2.75 | 3.45 | 2.71 | 3.04 | 6.77 |
| 25-34 years | 1.36 | 1.75 | 1.78 | 1.69 | 1.73 | 2.20 | 2.69 | 2.99 | 4.05 |
| 35-44 years | 1.28 | 1.42 | 1.51 | 1.68 | 1.91 | 2.08 | 2.23 | 2.47 | 3.20 |
| 45-54 years | 1.28 | 1.47 | 1.64 | 1.71 | 1.65 | 1.70 | 1.85 | 1.55 | 2.36 |
| 55-64 years | 1.10 | 1.12 | 1.14 | 1.15 | 1.17 | 1.17 | 1.30 | 1.17 | 1.18 |
| 65-74 years | 1.05 | 1.09 | 1.09 | 1.11 | 1.11 | 1.11 | 1.18 | 1.07 | 1.18 |
| 75-84 years | 0.88 | 0.87 | 0.87 | 0.87 | 0.93 | 0.94 | 1.02 | 1.03 | 1.07 |
| ≥85years | 0.88 | 0.83 | 0.85 | 0.87 | 0.93 | 0.98 | 1.06 | 1.18 | 1.22 |

**HFRS:** Hospital frailty risk score; **CCI:** Charlson Comorbidity Index

S2d Table. Logistics regression results (odds ratio) for 9 prediction periods of LOS and 8 age groups for CCI when combined with HFRS

| Subset data | **CCI (from HFRS+CCI models)** | | | | | | | | |
| --- | --- | --- | --- | --- | --- | --- | --- | --- | --- |
|  | **Length of Stay (LOS) group** | | | | | | | | |
|  | **LOS >3 days** | **LOS >7 days** | **LOS >10 days** | **LOS >14 days** | **LOS >21 days** | **LOS >30 days** | **LOS >45 days** | **LOS >60 days** | **LOS >90 days** |
| All ages | 1.37 | 1.33 | 1.31 | 1.27 | 1.23 | 1.18 | 1.13 | 1.09 | 1.04 |
| 16-24 years | 1.72 | 1.95 | 1.70 | 1.69 | 2.05 | 2.31 | 1.94 | 1.85 | 2.73 |
| 25-34 years | 1.40 | 1.41 | 1.38 | 1.34 | 1.41 | 1.40 | 1.47 | 1.30 | 1.37 |
| 35-44 years | 1.26 | 1.24 | 1.26 | 1.23 | 1.27 | 1.20 | 1.15 | 1.27 | 1.00 |
| 45-54 years | 1.28 | 1.29 | 1.28 | 1.26 | 1.23 | 1.19 | 1.17 | 1.09 | 1.00 |
| 55-64 years | 1.30 | 1.29 | 1.28 | 1.24 | 1.21 | 1.16 | 1.11 | 1.04 | 1.09 |
| 65-74 years | 1.35 | 1.32 | 1.29 | 1.26 | 1.21 | 1.16 | 1.15 | 1.16 | 1.17 |
| 75-84 years | 1.34 | 1.29 | 1.26 | 1.23 | 1.20 | 1.15 | 1.10 | 1.06 | 1.05 |
| ≥85years | 1.30 | 1.23 | 1.20 | 1.17 | 1.13 | 1.09 | 1.04 | 1.02 | 1.00 |

**HFRS:** Hospital frailty risk score; **CCI:** Charlson Comorbidity Index
