# Supplementary material for: Using the Hospital Frailty Risk Score to predict length of stay across all adult ages
Source: PLoS One. 2025 Jan 23;20(1):e0317234. doi: 10.1371/journal.pone.0317234 (PMC11756769; doi:10.1371/journal.pone.0317234)
Supplement: S3 Table — Logistics regression results (odds ratio) from models CCI alone or combined with one other variable (age, gender, HFRS) for each period of LOS and each age groups. (DOCX) [file pone.0317234.s003.docx]

**S3 Table: (S3a-S3d) Tables. Logistics regression results (odds ratio) from Models CCI alone or combined with one other variable (age, gender, HFRS) for each period of LOS for each age groups.**

S3a Table. Logistics regression results (odds ratio) for 9 prediction periods of LOS and 8 age groups for CCI alone

| Subset data | **CCI alone models** | | | | | | | | |
| --- | --- | --- | --- | --- | --- | --- | --- | --- | --- |
|  | **Length of Stay (LOS) group** | | | | | | | | |
|  | **LOS >3 days** | **LOS >7 days** | **LOS >10 days** | **LOS >14 days** | **LOS >21 days** | **LOS >30 days** | **LOS >45 days** | **LOS >60 days** | **LOS >90 days** |
| All ages | 1.58 | 1.56 | 1.53 | 1.50 | 1.45 | 1.40 | 1.36 | 1.32 | 1.27 |
| 16-24 years | 2.02 | 2.30 | 2.01 | 1.99 | 2.37 | 2.62 | 2.20 | 2.07 | 3.04 |
| 25-34 years | 1.69 | 1.65 | 1.60 | 1.56 | 1.62 | 1.61 | 1.67 | 1.51 | 1.56 |
| 35-44 years | 1.36 | 1.33 | 1.35 | 1.32 | 1.35 | 1.31 | 1.27 | 1.36 | 1.16 |
| 45-54 years | 1.41 | 1.42 | 1.41 | 1.39 | 1.37 | 1.34 | 1.33 | 1.27 | 1.09 |
| 55-64 years | 1.41 | 1.39 | 1.39 | 1.36 | 1.33 | 1.30 | 1.26 | 1.19 | 1.25 |
| 65-74 years | 1.47 | 1.45 | 1.42 | 1.40 | 1.36 | 1.32 | 1.32 | 1.32 | 1.33 |
| 75-84 years | 1.50 | 1.46 | 1.43 | 1.40 | 1.37 | 1.33 | 1.27 | 1.23 | 1.22 |
| ≥85years | 1.44 | 1.36 | 1.32 | 1.28 | 1.25 | 1.20 | 1.15 | 1.12 | 1.00 |

**HFRS:** Hospital frailty risk score; **CCI:** Charlson Comorbidity Index

S3b Table. Logistics regression results (odds ratio) for 9 prediction periods of LOS and 8 age groups for age when combined with CCI

| Subset data | **age (from CCI+age models)** | | | | | | | | |
| --- | --- | --- | --- | --- | --- | --- | --- | --- | --- |
|  | **Length of Stay (LOS) group** | | | | | | | | |
|  | **LOS >3 days** | **LOS >7 days** | **LOS >10 days** | **LOS >14 days** | **LOS >21 days** | **LOS >30 days** | **LOS >45 days** | **LOS >60 days** | **LOS >90 days** |
| All ages | 1.03 | 1.05 | 1.05 | 1.06 | 1.06 | 1.06 | 1.05 | 1.05 | 1.04 |
| 16-24 years | 1.03 | 1.04 | 1.04 | 1.05 | 1.10 | 1.14 | 1.16 | 1.27 | 1.28 |
| 25-34 years | 1.01 | 1.02 | 1.02 | 1.03 | 1.05 | 1.04 | 1.05 | 1.01 | 0.91 |
| 35-44 years | 1.02 | 1.02 | 1.01 | 1.02 | 1.02 | 1.02 | 1.03 | 0.98 | 0.91 |
| 45-54 years | 1.01 | 1.02 | 1.02 | 1.03 | 1.02 | 1.03 | 1.06 | 1.05 | 1.06 |
| 55-64 years | 1.04 | 1.05 | 1.06 | 1.06 | 1.06 | 1.05 | 1.05 | 1.05 | 1.06 |
| 65-74 years | 1.03 | 1.04 | 1.05 | 1.05 | 1.05 | 1.06 | 1.05 | 1.03 | 1.02 |
| 75-84 years | 1.06 | 1.08 | 1.08 | 1.08 | 1.08 | 1.08 | 1.09 | 1.08 | 1.09 |
| ≥85 years | 1.06 | 1.07 | 1.07 | 1.07 | 1.06 | 1.06 | 1.04 | 1.02 | 1.00 |

**HFRS:** Hospital frailty risk score; **CCI:** Charlson Comorbidity Index

S3c Table. Logistics regression results (odds ratio) for 9 prediction periods of LOS and 8 gender groups for gender when combined with CCI

| Subset data | **Gender (from CCI + gender models) Females Ref = 1** | | | | | | | | |
| --- | --- | --- | --- | --- | --- | --- | --- | --- | --- |
|  | **Length of Stay (LOS) group** | | | | | | | | |
|  | **LOS >3 days** | **LOS >7 days** | **LOS >10 days** | **LOS >14 days** | **LOS >21 days** | **LOS >30 days** | **LOS >45 days** | **LOS >60 days** | **LOS >90 days** |
| All ages | 1.02 | 0.99 | 0.98 | 0.98 | 1.01 | 1.05 | 1.15 | 1.19 | 1.36 |
| 16-24 years | 1.23 | 1.43 | 1.51 | 1.56 | 1.79 | 2.22 | 1.69 | 1.70 | 3.28 |
| 25-34 years | 1.26 | 1.53 | 1.52 | 1.40 | 1.38 | 1.69 | 1.93 | 2.37 | 3.36 |
| 35-44 years | 1.38 | 1.53 | 1.62 | 1.79 | 1.99 | 2.13 | 2.28 | 2.49 | 2.84 |
| 45-54 years | 1.37 | 1.58 | 1.76 | 1.84 | 1.76 | 1.81 | 1.93 | 1.65 | 2.49 |
| 55-64 years | 1.18 | 1.22 | 1.26 | 1.29 | 1.34 | 1.37 | 1.54 | 1.43 | 1.49 |
| 65-74 years | 1.05 | 1.08 | 1.09 | 1.12 | 1.14 | 1.15 | 1.23 | 1.14 | 1.26 |
| 75-84 years | 0.85 | 0.85 | 0.84 | 0.85 | 0.90 | 0.92 | 1.00 | 1.02 | 1.07 |
| ≥85 years | 0.83 | 0.78 | 0.80 | 0.83 | 0.88 | 0.93 | 1.01 | 1.13 | 1.16 |

**HFRS:** Hospital frailty risk score; **CCI:** Charlson Comorbidity Index

S3d Table. Logistics regression results (odds ratio) for 9 prediction periods of LOS and 8 age groups for HFRS when combined with CCI

| Subset data | **HFRS (from CCI+HFRS models)** | | | | | | | | |
| --- | --- | --- | --- | --- | --- | --- | --- | --- | --- |
|  | **Length of Stay (LOS) group** | | | | | | | | |
|  | **LOS >3 days** | **LOS >7 days** | **LOS >10 days** | **LOS >14 days** | **LOS >21 days** | **LOS >30 days** | **LOS >45 days** | **LOS >60 days** | **LOS >90 days** |
| All ages | 2.04 | 2.05 | 2.01 | 1.96 | 1.91 | 1.88 | 1.84 | 1.81 | 1.79 |
| 16-24 years | 2.09 | 2.15 | 2.19 | 2.28 | 2.28 | 2.15 | 2.22 | 2.29 | 2.17 |
| 25-34 years | 2.48 | 2.63 | 2.67 | 2.84 | 2.88 | 2.94 | 3.07 | 2.78 | 2.77 |
| 35-44 years | 2.45 | 2.47 | 2.38 | 2.49 | 2.53 | 2.62 | 2.61 | 2.64 | 3.09 |
| 45-54 years | 2.24 | 2.30 | 2.26 | 2.27 | 2.25 | 2.22 | 2.25 | 2.25 | 2.29 |
| 55-64 years | 2.21 | 2.21 | 2.18 | 2.15 | 2.12 | 2.13 | 2.14 | 2.13 | 2.18 |
| 65-74 years | 2.05 | 2.07 | 2.06 | 2.03 | 2.02 | 2.00 | 1.98 | 1.96 | 1.90 |
| 75-84 years | 1.78 | 1.79 | 1.76 | 1.73 | 1.72 | 1.70 | 1.67 | 1.67 | 1.66 |
| ≥85 years | 1.61 | 1.55 | 1.52 | 1.49 | 1.47 | 1.47 | 1.47 | 1.46 | 1.50 |

**HFRS:** Hospital frailty risk score; **CCI:** Charlson Comorbidity Index
