# Supplementary material for: Using the Hospital Frailty Risk Score to predict length of stay across all adult ages
Source: PLoS One. 2025 Jan 23;20(1):e0317234. doi: 10.1371/journal.pone.0317234 (PMC11756769; doi:10.1371/journal.pone.0317234)
Supplement: S4 Table — Area Under ROC for 9 periods of long length of stay and 8 age groups for models HFRS alone or combined with one other variable (age, gender, CCI). (DOCX) [file pone.0317234.s004.docx]

**S4 Table: (S4a-S4d) Tables. Area Under ROC for 9 periods of long length of stay and 8 age groups for** **models HFRS alone or combined with one other variable (age, gender, CCI).**

S4a Table. Area Under ROC for 9 periods of prediction long length of stay and 8 age groups for HFRS alone

| Subset data | **HFRS alone models** | | | | | | | | |
| --- | --- | --- | --- | --- | --- | --- | --- | --- | --- |
|  | **Length of Stay (LOS) group** | | | | | | | | |
|  | **LOS >3 days** | **LOS >7 days** | **LOS >10 days** | **LOS >14 days** | **LOS >21 days** | **LOS >30 days** | **LOS >45 days** | **LOS >60 days** | **LOS >90 days** |
| All ages | 0.779 | 0.827 | 0.841 | 0.853 | 0.867 | 0.875 | 0.880 | 0.880 | 0.890 |
| 16-24 years | 0.700 | 0.743 | 0.791 | 0.820 | 0.873 | 0.873 | 0.785 | 0.712 | 0.700 |
| 25-34 years | 0.714 | 0.774 | 0.774 | 0.815 | 0.841 | 0.847 | 0.840 | 0.833 | 0.730 |
| 35-44 years | 0.731 | 0.771 | 0.767 | 0.797 | 0.837 | 0.850 | 0.796 | 0.778 | 0.752 |
| 45-54 years | 0.734 | 0.791 | 0.799 | 0.823 | 0.848 | 0.854 | 0.862 | 0.866 | 0.900 |
| 55-64 years | 0.753 | 0.805 | 0.821 | 0.835 | 0.847 | 0.873 | 0.895 | 0.900 | 0.923 |
| 65-74 years | 0.747 | 0.797 | 0.814 | 0.832 | 0.853 | 0.862 | 0.888 | 0.898 | 0.898 |
| 75-84 years | 0.763 | 0.802 | 0.811 | 0.825 | 0.838 | 0.847 | 0.848 | 0.858 | 0.869 |
| ≥85years | 0.767 | 0.765 | 0.764 | 0.763 | 0.768 | 0.770 | 0.767 | 0.758 | 0.748 |

**HFRS:** Hospital frailty risk score; **CCI:** Charlson Comorbidity Index

S4b Table. Area Under ROC for 9 periods of prediction long length of stay and 8 age groups for HFRS combined with age

| Subset data | **HFRS+age models** | | | | | | | | |
| --- | --- | --- | --- | --- | --- | --- | --- | --- | --- |
|  | **Length of Stay (LOS) group** | | | | | | | | |
|  | **LOS >3 days** | **LOS >7 days** | **LOS >10 days** | **LOS >14 days** | **LOS >21 days** | **LOS >30 days** | **LOS >45 days** | **LOS >60 days** | **LOS >90 days** |
| All ages | 0.769 | 0.813 | 0.826 | 0.836 | 0.846 | 0.853 | 0.857 | 0.856 | 0.882 |
| 16-24 years | 0.681 | 0.739 | 0.766 | 0.802 | 0.859 | 0.840 | 0.720 | 0.729 | 0.875 |
| 25-34 years | 0.716 | 0.779 | 0.775 | 0.808 | 0.839 | 0.847 | 0.870 | 0.851 | 0.682 |
| 35-44 years | 0.728 | 0.769 | 0.764 | 0.797 | 0.835 | 0.852 | 0.791 | 0.769 | 0.834 |
| 45-54 years | 0.733 | 0.791 | 0.798 | 0.823 | 0.847 | 0.854 | 0.863 | 0.866 | 0.901 |
| 55-64 years | 0.749 | 0.795 | 0.811 | 0.824 | 0.841 | 0.870 | 0.894 | 0.899 | 0.923 |
| 65-74 years | 0.746 | 0.795 | 0.812 | 0.829 | 0.852 | 0.859 | 0.888 | 0.898 | 0.874 |
| 75-84 years | 0.759 | 0.796 | 0.805 | 0.818 | 0.830 | 0.841 | 0.842 | 0.855 | 0.864 |
| ≥85years | 0.763 | 0.762 | 0.761 | 0.760 | 0.766 | 0.767 | 0.764 | 0.758 | 0.741 |

**HFRS:** Hospital frailty risk score; **CCI:** Charlson Comorbidity Index

S4c Table. Area Under ROC for 9 periods of prediction long length of stay and 8 age groups for HFRS combined with gender

| Subset data | **HFRS + gender models** | | | | | | | | |
| --- | --- | --- | --- | --- | --- | --- | --- | --- | --- |
|  | **Length of Stay (LOS) group** | | | | | | | | |
|  | **LOS >3 days** | **LOS >7 days** | **LOS >10 days** | **LOS >14 days** | **LOS >21 days** | **LOS >30 days** | **LOS >45 days** | **LOS >60 days** | **LOS >90 days** |
| All ages | 0.779 | 0.827 | 0.841 | 0.853 | 0.865 | 0.871 | 0.878 | 0.874 | 0.888 |
| 16-24 years | 0.680 | 0.717 | 0.763 | 0.796 | 0.839 | 0.767 | 0.758 | 0.710 | 0.883 |
| 25-34 years | 0.709 | 0.768 | 0.775 | 0.815 | 0.840 | 0.836 | 0.881 | 0.904 | 0.797 |
| 35-44 years | 0.722 | 0.758 | 0.754 | 0.788 | 0.829 | 0.844 | 0.814 | 0.802 | 0.856 |
| 45-54 years | 0.728 | 0.786 | 0.793 | 0.820 | 0.842 | 0.853 | 0.865 | 0.857 | 0.857 |
| 55-64 years | 0.752 | 0.807 | 0.821 | 0.835 | 0.847 | 0.870 | 0.887 | 0.897 | 0.923 |
| 65-74 years | 0.747 | 0.797 | 0.815 | 0.832 | 0.852 | 0.862 | 0.889 | 0.900 | 0.890 |
| 75-84 years | 0.761 | 0.799 | 0.809 | 0.823 | 0.837 | 0.846 | 0.841 | 0.853 | 0.866 |
| ≥85years | 0.766 | 0.764 | 0.764 | 0.762 | 0.768 | 0.770 | 0.767 | 0.758 | 0.747 |

**HFRS:** Hospital frailty risk score; **CCI:** Charlson Comorbidity Index

S4d Table. Area Under ROC for 9 periods of prediction long length of stay and 8 age groups for HFRS combined with CCI

| Subset data | **HFRS+CCI models** | | | | | | | | |
| --- | --- | --- | --- | --- | --- | --- | --- | --- | --- |
|  | **Length of Stay (LOS) group** | | | | | | | | |
|  | **LOS >3 days** | **LOS >7 days** | **LOS >10 days** | **LOS >14 days** | **LOS >21 days** | **LOS >30 days** | **LOS >45 days** | **LOS >60 days** | **LOS >90 days** |
| All ages | 0.792 | 0.835 | 0.847 | 0.857 | 0.865 | 0.874 | 0.877 | 0.877 | 0.889 |
| 16-24 years | 0.681 | 0.740 | 0.795 | 0.819 | 0.866 | 0.797 | 0.492 | 0.424 | 0.522 |
| 25-34 years | 0.719 | 0.779 | 0.775 | 0.812 | 0.835 | 0.838 | 0.836 | 0.824 | 0.721 |
| 35-44 years | 0.735 | 0.776 | 0.773 | 0.802 | 0.837 | 0.841 | 0.781 | 0.760 | 0.760 |
| 45-54 years | 0.741 | 0.795 | 0.804 | 0.829 | 0.847 | 0.842 | 0.849 | 0.858 | 0.901 |
| 55-64 years | 0.765 | 0.813 | 0.831 | 0.844 | 0.852 | 0.869 | 0.893 | 0.901 | 0.923 |
| 65-74 years | 0.763 | 0.810 | 0.823 | 0.837 | 0.856 | 0.866 | 0.891 | 0.900 | 0.900 |
| 75-84 years | 0.776 | 0.812 | 0.818 | 0.827 | 0.838 | 0.845 | 0.841 | 0.854 | 0.866 |
| ≥85years | 0.779 | 0.773 | 0.771 | 0.767 | 0.771 | 0.771 | 0.768 | 0.755 | 0.727 |

**HFRS:** Hospital frailty risk score; **CCI:** Charlson Comorbidity Index
