# Supplementary material for: Using the Hospital Frailty Risk Score to predict length of stay across all adult ages
Source: PLoS One. 2025 Jan 23;20(1):e0317234. doi: 10.1371/journal.pone.0317234 (PMC11756769; doi:10.1371/journal.pone.0317234)
Supplement: S5 Table — Area Under ROC for 9 periods of long length of stay and 8 age groups for models CCI alone or combined with one other variable (age, gender, HFRS). (DOCX) [file pone.0317234.s005.docx]

**S5 Table:(S5a-S5d) Tables. Area Under ROC for 9 periods of long length of stay and 8 age groups for** **models CCI alone or combined with one other variable (age, gender, HFRS).**

S5a Table. Area Under ROC for 9 periods of prediction long length of stay and 8 age groups for CCI alone

| Subset data | **CCI alone models** | | | | | | | | |
| --- | --- | --- | --- | --- | --- | --- | --- | --- | --- |
|  | **Length of Stay (LOS) group** | | | | | | | | |
|  | **LOS >3 days** | **LOS >7 days** | **LOS >10 days** | **LOS >14 days** | **LOS >21 days** | **LOS >30 days** | **LOS >45 days** | **LOS >60 days** | **LOS >90 days** |
| All ages | 0.628 | 0.637 | 0.636 | 0.631 | 0.626 | 0.613 | 0.598 | 0.593 | 0.556 |
| 16-24 years | 0.536 | 0.554 | 0.556 | 0.549 | 0.566 | 0.609 | 0.431 | 0.426 | 0.530 |
| 25-34 years | 0.553 | 0.568 | 0.561 | 0.561 | 0.559 | 0.560 | 0.546 | 0.497 | 0.453 |
| 35-44 years | 0.567 | 0.572 | 0.575 | 0.588 | 0.581 | 0.550 | 0.507 | 0.536 | 0.570 |
| 45-54 years | 0.584 | 0.589 | 0.595 | 0.608 | 0.604 | 0.569 | 0.556 | 0.528 | 0.476 |
| 55-64 years | 0.609 | 0.620 | 0.628 | 0.627 | 0.627 | 0.610 | 0.598 | 0.585 | 0.608 |
| 65-74 years | 0.627 | 0.633 | 0.629 | 0.626 | 0.620 | 0.619 | 0.627 | 0.617 | 0.599 |
| 75-84 years | 0.633 | 0.638 | 0.633 | 0.624 | 0.615 | 0.604 | 0.575 | 0.552 | 0.529 |
| ≥85years | 0.620 | 0.612 | 0.607 | 0.593 | 0.582 | 0.574 | 0.563 | 0.558 | 0.501 |

**HFRS:** Hospital frailty risk score; **CCI:** Charlson Comorbidity Index

S5b Table. Area Under ROC for 9 periods of prediction long length of stay and 8 age groups for CCI combined with age

| Subset data | **CCI+age models** | | | | | | | | |
| --- | --- | --- | --- | --- | --- | --- | --- | --- | --- |
|  | **Length of Stay (LOS) group** | | | | | | | | |
|  | **LOS >3 days** | **LOS >7 days** | **LOS >10 days** | **LOS >14 days** | **LOS >21 days** | **LOS >30 days** | **LOS >45 days** | **LOS >60 days** | **LOS >90 days** |
| All ages | 0.703 | 0.743 | 0.756 | 0.762 | 0.762 | 0.755 | 0.741 | 0.720 | 0.693 |
| 16-24 years | 0.539 | 0.566 | 0.602 | 0.603 | 0.642 | 0.711 | 0.528 | 0.670 | 0.513 |
| 25-34 years | 0.562 | 0.584 | 0.579 | 0.578 | 0.576 | 0.566 | 0.605 | 0.475 | 0.549 |
| 35-44 years | 0.573 | 0.582 | 0.571 | 0.596 | 0.579 | 0.547 | 0.541 | 0.541 | 0.798 |
| 45-54 years | 0.589 | 0.599 | 0.607 | 0.631 | 0.621 | 0.582 | 0.583 | 0.558 | 0.536 |
| 55-64 years | 0.617 | 0.627 | 0.628 | 0.630 | 0.634 | 0.613 | 0.626 | 0.612 | 0.620 |
| 65-74 years | 0.637 | 0.647 | 0.644 | 0.646 | 0.644 | 0.639 | 0.649 | 0.629 | 0.586 |
| 75-84 years | 0.652 | 0.666 | 0.662 | 0.653 | 0.644 | 0.636 | 0.617 | 0.582 | 0.558 |
| ≥85years | 0.655 | 0.649 | 0.647 | 0.634 | 0.625 | 0.611 | 0.592 | 0.581 | 0.485 |

**HFRS:** Hospital frailty risk score; **CCI:** Charlson Comorbidity Index

S5c Table. Area Under ROC for 9 periods of prediction long length of stay and 8 age groups for CCI combined with gender

| Subset data | **CCI + gender models** | | | | | | | | |
| --- | --- | --- | --- | --- | --- | --- | --- | --- | --- |
|  | **Length of Stay (LOS) group** | | | | | | | | |
|  | **LOS >3 days** | **LOS >7 days** | **LOS >10 days** | **LOS >14 days** | **LOS >21 days** | **LOS >30 days** | **LOS >45 days** | **LOS >60 days** | **LOS >90 days** |
| All ages | 0.632 | 0.641 | 0.640 | 0.635 | 0.633 | 0.630 | 0.607 | 0.608 | 0.593 |
| 16-24 years | 0.554 | 0.566 | 0.581 | 0.583 | 0.588 | 0.626 | 0.441 | 0.451 | 0.541 |
| 25-34 years | 0.578 | 0.616 | 0.617 | 0.607 | 0.598 | 0.620 | 0.606 | 0.723 | 0.662 |
| 35-44 years | 0.586 | 0.585 | 0.596 | 0.613 | 0.619 | 0.630 | 0.635 | 0.643 | 0.742 |
| 45-54 years | 0.600 | 0.626 | 0.642 | 0.660 | 0.655 | 0.642 | 0.666 | 0.618 | 0.620 |
| 55-64 years | 0.611 | 0.625 | 0.628 | 0.630 | 0.632 | 0.617 | 0.593 | 0.568 | 0.623 |
| 65-74 years | 0.634 | 0.643 | 0.638 | 0.637 | 0.626 | 0.620 | 0.624 | 0.624 | 0.615 |
| 75-84 years | 0.641 | 0.645 | 0.640 | 0.629 | 0.619 | 0.608 | 0.569 | 0.549 | 0.552 |
| ≥85years | 0.632 | 0.626 | 0.621 | 0.606 | 0.591 | 0.582 | 0.562 | 0.568 | 0.508 |

**HFRS:** Hospital frailty risk score; **CCI:** Charlson Comorbidity Index

S5d Table. Area Under ROC for 9 periods of prediction long length of stay and 8 age groups for CCI combined with HFRS

| Subset data | **CCI+HFRS models** | | | | | | | | |
| --- | --- | --- | --- | --- | --- | --- | --- | --- | --- |
|  | **Length of Stay (LOS) group** | | | | | | | | |
|  | **LOS >3 days** | **LOS >7 days** | **LOS >10 days** | **LOS >14 days** | **LOS >21 days** | **LOS >30 days** | **LOS >45 days** | **LOS >60 days** | **LOS >90 days** |
| All ages | 0.792 | 0.835 | 0.847 | 0.857 | 0.865 | 0.874 | 0.877 | 0.877 | 0.889 |
| 16-24 years | 0.681 | 0.740 | 0.795 | 0.819 | 0.866 | 0.797 | 0.492 | 0.424 | 0.522 |
| 25-34 years | 0.719 | 0.779 | 0.775 | 0.812 | 0.835 | 0.838 | 0.836 | 0.824 | 0.721 |
| 35-44 years | 0.735 | 0.776 | 0.773 | 0.802 | 0.837 | 0.841 | 0.781 | 0.760 | 0.760 |
| 45-54 years | 0.741 | 0.795 | 0.804 | 0.829 | 0.847 | 0.842 | 0.849 | 0.858 | 0.901 |
| 55-64 years | 0.765 | 0.813 | 0.831 | 0.844 | 0.852 | 0.869 | 0.893 | 0.901 | 0.923 |
| 65-74 years | 0.763 | 0.810 | 0.823 | 0.837 | 0.856 | 0.866 | 0.891 | 0.900 | 0.889 |
| 75-84 years | 0.776 | 0.812 | 0.818 | 0.827 | 0.838 | 0.845 | 0.841 | 0.854 | 0.866 |
| ≥85years | 0.779 | 0.773 | 0.771 | 0.767 | 0.771 | 0.771 | 0.768 | 0.755 | 0.727 |

**HFRS:** Hospital frailty risk score; **CCI:** Charlson Comorbidity Index
