# Supplementary material for: Using the Hospital Frailty Risk Score to predict length of stay across all adult ages
Source: PLoS One. 2025 Jan 23;20(1):e0317234. doi: 10.1371/journal.pone.0317234 (PMC11756769; doi:10.1371/journal.pone.0317234)
Supplement: S6 Table — Area Under ROC for 9 periods of long length of stay for models HFRS alone or combined with one other variable and models CCI alone or combined with one other variable for all admissions, non-elective admissions, and elective admissions. (DOCX) [file pone.0317234.s006.docx]

**S6 Table: (S6a-S6f) Tables.** **Area Under ROC for 9 periods of long length of stay for all patients, elective patients, and non-elective patients.**

S6a Table. Area Under ROC for 9 periods of prediction long length of stay and all patients for HFRS models

| All admissions | **LOS > 3 days** | **LOS > 7 days** | **LOS >10 days** | **LOS> 14 days** | **LOS > 21 days** | **LOS > 30 days** | **LOS > 45 days** | **LOS > 60 days** | **LOS > 90 days** |
| --- | --- | --- | --- | --- | --- | --- | --- | --- | --- |
| HFRS alone | 0.779 | 0.827 | 0.841 | 0.853 | 0.867 | 0.875 | 0.880 | 0.880 | 0.890 |
| HFRS+Age | 0.769 | 0.813 | 0.826 | 0.836 | 0.846 | 0.853 | 0.857 | 0.856 | 0.882 |
| HFRS+Gender | 0.779 | 0.827 | 0.841 | 0.853 | 0.865 | 0.871 | 0.878 | 0.874 | 0.888 |
| HFRS+CCI | 0.792 | 0.835 | 0.847 | 0.857 | 0.865 | 0.874 | 0.877 | 0.877 | 0.889 |

S6b Table. Area Under ROC for 9 periods of prediction long length of stay and non-elective patients for HFRS models

| Non-elective admissions | **LOS > 3 days** | **LOS > 7 days** | **LOS >10 days** | **LOS> 14 days** | **LOS > 21 days** | **LOS > 30 days** | **LOS > 45 days** | **LOS > 60 days** | **LOS > 90 days** |
| --- | --- | --- | --- | --- | --- | --- | --- | --- | --- |
| HFRS alone | 0.714 | 0.752 | 0.765 | 0.776 | 0.792 | 0.797 | 0.802 | 0.803 | 0.814 |
| HFRS+Age | 0.741 | 0.772 | 0.781 | 0.787 | 0.790 | 0.791 | 0.789 | 0.785 | 0.796 |
| HFRS+Gender | 0.712 | 0.753 | 0.763 | 0.774 | 0.787 | 0.794 | 0.797 | 0.797 | 0.808 |
| HFRS+CCI | 0.728 | 0.761 | 0.772 | 0.780 | 0.790 | 0.795 | 0.800 | 0.801 | 0.812 |

S6c Table. Area Under ROC for 9 periods of prediction long length of stay and elective patients for HFRS models

| Elective admissions | **LOS > 3 days** | **LOS > 7 days** | **LOS >10 days** | **LOS> 14 days** | **LOS > 21 days** | **LOS > 30 days** | **LOS > 45 days** | **LOS > 60 days** | **LOS > 90 days** |
| --- | --- | --- | --- | --- | --- | --- | --- | --- | --- |
| HFRS alone | 0.647 | 0.756 | 0.788 | 0.817 | 0.856 | 0.872 | 0.889 | 0.901 | 0.911 |
| HFRS+Age | 0.642 | 0.739 | 0.767 | 0.791 | 0.827 | 0.847 | 0.865 | 0.885 | 0.902 |
| HFRS+Gender | 0.646 | 0.756 | 0.788 | 0.817 | 0.856 | 0.872 | 0.885 | 0.895 | 0.911 |
| HFRS+CCI | 0.668 | 0.766 | 0.794 | 0.818 | 0.852 | 0.868 | 0.886 | 0.897 | 0.910 |

S6d Table. Area Under ROC for 9 periods of prediction long length of stay and all patients for CCI models

| All admissions | **LOS > 3 days** | **LOS > 7 days** | **LOS >10 days** | **LOS> 14 days** | **LOS > 21 days** | **LOS > 30 days** | **LOS > 45 days** | **LOS > 60 days** | **LOS > 90 days** |
| --- | --- | --- | --- | --- | --- | --- | --- | --- | --- |
| CCI alone | 0.628 | 0.637 | 0.636 | 0.631 | 0.626 | 0.613 | 0.598 | 0.593 | 0.556 |
| CCI+Age | 0.703 | 0.743 | 0.756 | 0.762 | 0.762 | 0.755 | 0.741 | 0.720 | 0.693 |
| CCI+Gender | 0.632 | 0.641 | 0.640 | 0.635 | 0.633 | 0.620 | 0.607 | 0.608 | 0.593 |
| CCI+HFRS | 0.792 | 0.835 | 0.847 | 0.857 | 0.865 | 0.874 | 0.877 | 0.877 | 0.889 |

S6e Table. Area Under ROC for 9 periods of prediction long length of stay and non-elective patients for CCI models

| Non-elective admissions | **LOS > 3 days** | **LOS > 7 days** | **LOS >10 days** | **LOS> 14 days** | **LOS > 21 days** | **LOS > 30 days** | **LOS > 45 days** | **LOS > 60 days** | **LOS > 90 days** |
| --- | --- | --- | --- | --- | --- | --- | --- | --- | --- |
| CCI alone | 0.597 | 0.600 | 0.599 | 0.594 | 0.588 | 0.573 | 0.556 | 0.544 | 0.525 |
| CCI+Age | 0.721 | 0.743 | 0.749 | 0.749 | 0.744 | 0.732 | 0.716 | 0.693 | 0.654 |
| CCI+Gender | 0.606 | 0.604 | 0.595 | 0.591 | 0.593 | 0.579 | 0.571 | 0.563 | 0.553 |
| CCI+HFRS | 0.728 | 0.761 | 0.772 | 0.780 | 0.790 | 0.795 | 0.800 | 0.801 | 0.812 |

S6f Table. Area Under ROC for 9 periods of prediction long length of stay and elective patients for CCI models

| Elective admissions | **LOS > 3 days** | **LOS > 7 days** | **LOS >10 days** | **LOS> 14 days** | **LOS > 21 days** | **LOS > 30 days** | **LOS > 45 days** | **LOS > 60 days** | **LOS > 90 days** |
| --- | --- | --- | --- | --- | --- | --- | --- | --- | --- |
| CCI alone | 0.583 | 0.608 | 0.614 | 0.615 | 0.623 | 0.626 | 0.637 | 0.620 | 0.570 |
| CCI+Age | 0.613 | 0.661 | 0.675 | 0.683 | 0.684 | 0.677 | 0.685 | 0.700 | 0.708 |
| CCI+Gender | 0.587 | 0.616 | 0.625 | 0.625 | 0.637 | 0.643 | 0.651 | 0.646 | 0.635 |
| CCI+HFRS | 0.668 | 0.766 | 0.794 | 0.818 | 0.852 | 0.868 | 0.886 | 0.897 | 0.915 |
