# Supplementary material for: Using the Hospital Frailty Risk Score to predict length of stay across all adult ages
Source: PLoS One. 2025 Jan 23;20(1):e0317234. doi: 10.1371/journal.pone.0317234 (PMC11756769; doi:10.1371/journal.pone.0317234)
Supplement: S7 Table — Area Under ROC for 9 periods of long length of stay and 8 age groups for models HFRS alone or combined with one other variable (age, gender, CCI) for non-elective admissions. (DOCX) [file pone.0317234.s007.docx]

**S7 Table:(S7a-S7d) Tables. Area Under ROC for 9 periods of long length of stay and 8 age groups for** **models HFRS alone or combined with one other variable (age, gender, CCI) for non-elective admissions.**

S7a Table. Area Under ROC for 9 periods of prediction long length of stay and 8 age groups for HFRS alone

| Subset data | **HFRS alone models** | | | | | | | | |
| --- | --- | --- | --- | --- | --- | --- | --- | --- | --- |
|  | **Length of Stay (LOS) group** | | | | | | | | |
|  | **LOS >3 days** | **LOS >7 days** | **LOS >10 days** | **LOS >14 days** | **LOS >21 days** | **LOS >30 days** | **LOS >45 days** | **LOS >60 days** | **LOS >90 days** |
| 16-24 years | 0.626 | 0.694 | 0.723 | 0.772 | 0.814 | 0.798 | 0.859 | 0.862 | 0.898 |
| 25-34 years | 0.671 | 0.724 | 0.737 | 0.775 | 0.813 | 0.827 | 0.866 | 0.861 | 0.857 |
| 35-44 years | 0.665 | 0.705 | 0.720 | 0.749 | 0.769 | 0.788 | 0.782 | 0.804 | 0.875 |
| 45-54 years | 0.657 | 0.705 | 0.721 | 0.741 | 0.760 | 0.771 | 0.804 | 0.818 | 0.846 |
| 55-64 years | 0.659 | 0.695 | 0.713 | 0.73 | 0.748 | 0.767 | 0.791 | 0.801 | 0.848 |
| 65-74 years | 0.651 | 0.684 | 0.702 | 0.717 | 0.741 | 0.755 | 0.771 | 0.781 | 0.795 |
| 75-84 years | 0.641 | 0.676 | 0.686 | 0.696 | 0.711 | 0.721 | 0.721 | 0.732 | 0.751 |
| ≥85 years | 0.617 | 0.634 | 0.638 | 0.644 | 0.653 | 0.664 | 0.671 | 0.669 | 0.692 |

**HFRS:** Hospital frailty risk score; **CCI:** Charlson Comorbidity Index

S7b Table. Area Under ROC for 9 periods of prediction long length of stay and 8 age groups for HFRS combined with age

| Subset data | **HFRS+age models** | | | | | | | | |
| --- | --- | --- | --- | --- | --- | --- | --- | --- | --- |
|  | **Length of Stay (LOS) group** | | | | | | | | |
|  | **LOS >3 days** | **LOS >7 days** | **LOS >10 days** | **LOS >14 days** | **LOS >21 days** | **LOS >30 days** | **LOS >45 days** | **LOS >60 days** | **LOS >90 days** |
| 16-24 years | 0.629 | 0.698 | 0.726 | 0.775 | 0.821 | 0.812 | 0.862 | 0.857 | 0.873 |
| 25-34 years | 0.669 | 0.719 | 0.734 | 0.772 | 0.811 | 0.828 | 0.861 | 0.857 | 0.849 |
| 35-44 years | 0.663 | 0.700 | 0.717 | 0.749 | 0.770 | 0.792 | 0.788 | 0.797 | 0.856 |
| 45-54 years | 0.656 | 0.701 | 0.715 | 0.734 | 0.755 | 0.765 | 0.799 | 0.811 | 0.832 |
| 55-64 years | 0.656 | 0.690 | 0.709 | 0.723 | 0.743 | 0.764 | 0.790 | 0.802 | 0.848 |
| 65-74 years | 0.650 | 0.681 | 0.699 | 0.714 | 0.739 | 0.752 | 0.771 | 0.779 | 0.794 |
| 75-84 years | 0.641 | 0.674 | 0.684 | 0.693 | 0.708 | 0.719 | 0.718 | 0.731 | 0.750 |
| ≥85 years | 0.617 | 0.633 | 0.637 | 0.643 | 0.652 | 0.663 | 0.671 | 0.671 | 0.701 |

**HFRS:** Hospital frailty risk score; **CCI:** Charlson Comorbidity Index

S7c Table. Area Under ROC for 9 periods of prediction long length of stay and 8 age groups for HFRS combined with gender

| Subset data | **HFRS + gender models** | | | | | | | | |
| --- | --- | --- | --- | --- | --- | --- | --- | --- | --- |
|  | **Length of Stay (LOS) group** | | | | | | | | |
|  | **LOS >3 days** | **LOS >7 days** | **LOS >10 days** | **LOS >14 days** | **LOS >21 days** | **LOS >30 days** | **LOS >45 days** | **LOS >60 days** | **LOS >90 days** |
| 16-24 years | 0.64 | 0.707 | 0.732 | 0.773 | 0.822 | 0.808 | 0.829 | 0.820 | 0.877 |
| 25-34 years | 0.68 | 0.732 | 0.745 | 0.779 | 0.816 | 0.836 | 0.860 | 0.843 | 0.880 |
| 35-44 years | 0.666 | 0.704 | 0.713 | 0.739 | 0.761 | 0.783 | 0.784 | 0.801 | 0.891 |
| 45-54 years | 0.657 | 0.703 | 0.717 | 0.736 | 0.753 | 0.766 | 0.791 | 0.812 | 0.810 |
| 55-64 years | 0.658 | 0.694 | 0.712 | 0.730 | 0.748 | 0.767 | 0.786 | 0.802 | 0.848 |
| 65-74 years | 0.651 | 0.684 | 0.702 | 0.717 | 0.741 | 0.755 | 0.771 | 0.781 | 0.797 |
| 75-84 years | 0.641 | 0.675 | 0.686 | 0.696 | 0.711 | 0.721 | 0.720 | 0.731 | 0.750 |
| ≥85 years | 0.617 | 0.634 | 0.638 | 0.644 | 0.653 | 0.663 | 0.669 | 0.666 | 0.688 |

**HFRS:** Hospital frailty risk score; **CCI:** Charlson Comorbidity Index

S7d Table. Area Under ROC for 9 periods of prediction long length of stay and 8 age groups for HFRS combined with CCI

| Subset data | **HFRS+CCI models** | | | | | | | | |
| --- | --- | --- | --- | --- | --- | --- | --- | --- | --- |
|  | **Length of Stay (LOS) group** | | | | | | | | |
|  | **LOS >3 days** | **LOS >7 days** | **LOS >10 days** | **LOS >14 days** | **LOS >21 days** | **LOS >30 days** | **LOS >45 days** | **LOS >60 days** | **LOS >90 days** |
| 16-24 years | 0.627 | 0.694 | 0.725 | 0.766 | 0.807 | 0.796 | 0.840 | 0.843 | 0.880 |
| 25-34 years | 0.676 | 0.73 | 0.742 | 0.777 | 0.816 | 0.828 | 0.864 | 0.861 | 0.862 |
| 35-44 years | 0.672 | 0.711 | 0.726 | 0.756 | 0.775 | 0.788 | 0.778 | 0.799 | 0.878 |
| 45-54 years | 0.664 | 0.710 | 0.725 | 0.746 | 0.763 | 0.771 | 0.801 | 0.818 | 0.841 |
| 55-64 years | 0.669 | 0.703 | 0.720 | 0.734 | 0.748 | 0.765 | 0.791 | 0.803 | 0.848 |
| 65-74 years | 0.659 | 0.689 | 0.705 | 0.718 | 0.741 | 0.755 | 0.771 | 0.781 | 0.795 |
| 75-84 years | 0.647 | 0.679 | 0.688 | 0.696 | 0.710 | 0.720 | 0.721 | 0.733 | 0.751 |
| ≥85 years | 0.624 | 0.637 | 0.64 | 0.645 | 0.653 | 0.664 | 0.671 | 0.667 | 0.698 |

**HFRS:** Hospital frailty risk score; **CCI:** Charlson Comorbidity Index
