# Supplementary material for: Using the Hospital Frailty Risk Score to predict length of stay across all adult ages
Source: PLoS One. 2025 Jan 23;20(1):e0317234. doi: 10.1371/journal.pone.0317234 (PMC11756769; doi:10.1371/journal.pone.0317234)
Supplement: S8 Table — Area Under ROC for 9 periods of long length of stay and 8 age groups for models CCI alone or combined with one other variable (age, gender, HFRS) for non-elective admissions. (DOCX) [file pone.0317234.s008.docx]

**S8 (S8a-S8d) Table. Area Under ROC for 9 periods of long length of stay and 8 age groups for** **models CCI alone or combined with one other variable (age, gender, HFRS) for non-elective admissions.**

S8a Table. Area Under ROC for 9 periods of prediction long length of stay and 8 age groups for CCI alone and non-elective admissions.

| Subset data | **CCI alone models** | | | | | | | | |
| --- | --- | --- | --- | --- | --- | --- | --- | --- | --- |
|  | **Length of Stay (LOS) group** | | | | | | | | |
|  | **LOS >3 days** | **LOS >7 days** | **LOS >10 days** | **LOS >14 days** | **LOS >21 days** | **LOS >30 days** | **LOS >45 days** | **LOS >60 days** | **LOS >90 days** |
| 16-24 years | 0.525 | 0.534 | 0.530 | 0.520 | 0.537 | 0.557 | 0.495 | 0.474 | 0.496 |
| 25-34 years | 0.544 | 0.556 | 0.556 | 0.563 | 0.581 | 0.582 | 0.596 | 0.584 | 0.606 |
| 35-44 years | 0.551 | 0.550 | 0.557 | 0.562 | 0.557 | 0.538 | 0.535 | 0.563 | 0.527 |
| 45-54 years | 0.557 | 0.562 | 0.565 | 0.57 | 0.569 | 0.552 | 0.551 | 0.532 | 0.491 |
| 55-64 years | 0.562 | 0.561 | 0.562 | 0.555 | 0.547 | 0.532 | 0.523 | 0.510 | 0.528 |
| 65-74 years | 0.559 | 0.556 | 0.553 | 0.546 | 0.538 | 0.524 | 0.526 | 0.524 | 0.521 |
| 75-84 years | 0.554 | 0.553 | 0.552 | 0.549 | 0.542 | 0.526 | 0.503 | 0.49 | 0.489 |
| ≥85 years | 0.545 | 0.54 | 0.535 | 0.528 | 0.522 | 0.508 | 0.513 | 0.524 | 0.575 |

**HFRS:** Hospital frailty risk score; **CCI:** Charlson Comorbidity Index

S8b Table. Area Under ROC for 9 periods of prediction long length of stay and 8 age groups for CCI combined with age and non-elective admissions.

| Subset data | **CCI+age models** | | | | | | | | |
| --- | --- | --- | --- | --- | --- | --- | --- | --- | --- |
|  | **Length of Stay (LOS) group** | | | | | | | | |
|  | **LOS >3 days** | **LOS >7 days** | **LOS >10 days** | **LOS >14 days** | **LOS >21 days** | **LOS >30 days** | **LOS >45 days** | **LOS >60 days** | **LOS >90 days** |
| 16-24 years | 0.547 | 0.562 | 0.565 | 0.566 | 0.595 | 0.631 | 0.581 | 0.634 | 0.615 |
| 25-34 years | 0.563 | 0.578 | 0.574 | 0.588 | 0.619 | 0.613 | 0.637 | 0.606 | 0.644 |
| 35-44 years | 0.579 | 0.579 | 0.580 | 0.584 | 0.580 | 0.565 | 0.571 | 0.556 | 0.561 |
| 45-54 years | 0.572 | 0.582 | 0.584 | 0.595 | 0.589 | 0.58 | 0.595 | 0.583 | 0.566 |
| 55-64 years | 0.582 | 0.585 | 0.588 | 0.586 | 0.576 | 0.562 | 0.554 | 0.528 | 0.561 |
| 65-74 years | 0.572 | 0.575 | 0.573 | 0.572 | 0.562 | 0.557 | 0.550 | 0.531 | 0.481 |
| 75-84 years | 0.566 | 0.573 | 0.574 | 0.572 | 0.567 | 0.556 | 0.542 | 0.531 | 0.537 |
| ≥85 years | 0.555 | 0.559 | 0.557 | 0.551 | 0.544 | 0.532 | 0.512 | 0.515 | 0.585 |

**HFRS:** Hospital frailty risk score; **CCI:** Charlson Comorbidity Index

S8c Table. Area Under ROC for 9 periods of prediction long length of stay and 8 age groups for CCI combined with gender and non-elective admissions.

| Subset data | **CCI + gender models** | | | | | | | | |
| --- | --- | --- | --- | --- | --- | --- | --- | --- | --- |
|  | **Length of Stay (LOS) group** | | | | | | | | |
|  | **LOS >3 days** | **LOS >7 days** | **LOS >10 days** | **LOS >14 days** | **LOS >21 days** | **LOS >30 days** | **LOS >45 days** | **LOS >60 days** | **LOS >90 days** |
| 16-24 years | 0.564 | 0.595 | 0.598 | 0.593 | 0.625 | 0.665 | 0.593 | 0.585 | 0.667 |
| 25-34 years | 0.587 | 0.611 | 0.610 | 0.606 | 0.616 | 0.637 | 0.660 | 0.665 | 0.709 |
| 35-44 years | 0.580 | 0.585 | 0.597 | 0.599 | 0.607 | 0.603 | 0.619 | 0.634 | 0.633 |
| 45-54 years | 0.563 | 0.575 | 0.590 | 0.596 | 0.592 | 0.587 | 0.594 | 0.571 | 0.566 |
| 55-64 years | 0.564 | 0.562 | 0.559 | 0.560 | 0.553 | 0.545 | 0.540 | 0.523 | 0.539 |
| 65-74 years | 0.561 | 0.557 | 0.553 | 0.544 | 0.536 | 0.524 | 0.536 | 0.524 | 0.518 |
| 75-84 years | 0.556 | 0.561 | 0.559 | 0.555 | 0.543 | 0.527 | 0.51 | 0.502 | 0.498 |
| ≥85 years | 0.548 | 0.551 | 0.546 | 0.538 | 0.524 | 0.509 | 0.522 | 0.539 | 0.579 |

**HFRS:** Hospital frailty risk score; **CCI:** Charlson Comorbidity Index

S8d Table. Area Under ROC for 9 periods of prediction long length of stay and 8 age groups for CCI combined with HFRS and non-elective admissions.

| Subset data | **CCI+HFRS models** | | | | | | | | |
| --- | --- | --- | --- | --- | --- | --- | --- | --- | --- |
|  | **Length of Stay (LOS) group** | | | | | | | | |
|  | **LOS >3 days** | **LOS >7 days** | **LOS >10 days** | **LOS >14 days** | **LOS >21 days** | **LOS >30 days** | **LOS >45 days** | **LOS >60 days** | **LOS >90 days** |
| 16-24 years | 0.627 | 0.694 | 0.725 | 0.766 | 0.807 | 0.796 | 0.840 | 0.843 | 0.880 |
| 25-34 years | 0.676 | 0.730 | 0.742 | 0.777 | 0.816 | 0.828 | 0.865 | 0.861 | 0.862 |
| 35-44 years | 0.672 | 0.711 | 0.726 | 0.756 | 0.775 | 0.788 | 0.778 | 0.799 | 0.878 |
| 45-54 years | 0.664 | 0.710 | 0.725 | 0.746 | 0.763 | 0.771 | 0.801 | 0.818 | 0.841 |
| 55-64 years | 0.669 | 0.703 | 0.720 | 0.734 | 0.748 | 0.765 | 0.791 | 0.803 | 0.848 |
| 65-74 years | 0.659 | 0.689 | 0.705 | 0.718 | 0.741 | 0.755 | 0.771 | 0.781 | 0.795 |
| 75-84 years | 0.647 | 0.679 | 0.688 | 0.696 | 0.71 | 0.72 | 0.721 | 0.733 | 0.751 |
| ≥85 years | 0.624 | 0.637 | 0.64 | 0.645 | 0.653 | 0.664 | 0.671 | 0.667 | 0.698 |

**HFRS:** Hospital frailty risk score; **CCI:** Charlson Comorbidity Index
