# Supplementary material for: Using the Hospital Frailty Risk Score to predict length of stay across all adult ages
Source: PLoS One. 2025 Jan 23;20(1):e0317234. doi: 10.1371/journal.pone.0317234 (PMC11756769; doi:10.1371/journal.pone.0317234)
Supplement: S9 Table — Area Under ROC for 9 periods of long length of stay and 8 age groups for models HFRS alone or combined with one other variable (age, gender, CCI) for elective admissions. (DOCX) [file pone.0317234.s009.docx]

**S9 Table: (S9a-S9d) Tables. Area Under ROC for 9 periods of long length of stay and 8 age groups for** **models HFRS alone or combined with one other variable (age, gender, CCI) for elective admissions.**

S9a Table. Area Under ROC for 9 periods of prediction long length of stay and 8 age groups for HFRS alone and elective admissions.

| Subset data | **HFRS alone models** | | | | | | | | |
| --- | --- | --- | --- | --- | --- | --- | --- | --- | --- |
|  | **Length of Stay (LOS) group** | | | | | | | | |
|  | **LOS >3 days** | **LOS >7 days** | **LOS >10 days** | **LOS >14 days** | **LOS >21 days** | **LOS >30 days** | **LOS >45 days** | **LOS >60 days** | **LOS >90 days** |
| 16-24 years | 0.685 | 0.759 | 0.757 | 0.777 | 0.809 | 0.765 | 0.740 | 0.668 | NA |
| 25-34 years | 0.682 | 0.786 | 0.806 | 0.820 | 0.796 | 0.804 | 0.911 | 0.879 | NA |
| 35-44 years | 0.669 | 0.760 | 0.766 | 0.790 | 0.858 | 0.901 | 0.795 | 0.710 | NA |
| 45-54 years | 0.634 | 0.754 | 0.770 | 0.792 | 0.854 | 0.860 | 0.856 | 0.841 | 0.937 |
| 55-64 years | 0.646 | 0.753 | 0.775 | 0.796 | 0.838 | 0.852 | 0.875 | 0.905 | 0.866 |
| 65-74 years | 0.620 | 0.733 | 0.770 | 0.798 | 0.835 | 0.861 | 0.900 | 0.904 | 0.899 |
| 75-84 years | 0.624 | 0.724 | 0.761 | 0.803 | 0.845 | 0.862 | 0.861 | 0.896 | 0.893 |
| ≥85 years | 0.694 | 0.770 | 0.806 | 0.827 | 0.859 | 0.883 | 0.921 | 0.921 | 0.924 |

**HFRS:** Hospital frailty risk score; **CCI:** Charlson Comorbidity Index

S9b Table. Area Under ROC for 9 periods of prediction long length of stay and 8 age groups for HFRS combined with age and elective admissions.

| Subset data | **HFRS+age models** | | | | | | | | |
| --- | --- | --- | --- | --- | --- | --- | --- | --- | --- |
|  | **Length of Stay (LOS) group** | | | | | | | | |
|  | **LOS >3 days** | **LOS >7 days** | **LOS >10 days** | **LOS >14 days** | **LOS >21 days** | **LOS >30 days** | **LOS >45 days** | **LOS >60 days** | **LOS >90 days** |
| 16-24 years | 0.683 | 0.724 | 0.715 | 0.722 | 0.793 | 0.732 | 0.733 | 0.649 | NA |
| 25-34 years | 0.683 | 0.783 | 0.803 | 0.819 | 0.780 | 0.784 | 0.898 | 0.985 | NA |
| 35-44 years | 0.675 | 0.757 | 0.762 | 0.784 | 0.840 | 0.884 | 0.818 | 0.874 | NA |
| 45-54 years | 0.635 | 0.756 | 0.768 | 0.785 | 0.837 | 0.853 | 0.832 | 0.866 | 0.936 |
| 55-64 years | 0.645 | 0.752 | 0.777 | 0.796 | 0.840 | 0.841 | 0.873 | 0.922 | 0.900 |
| 65-74 years | 0.623 | 0.732 | 0.771 | 0.797 | 0.828 | 0.858 | 0.893 | 0.897 | 0.892 |
| 75-84 years | 0.616 | 0.715 | 0.753 | 0.795 | 0.845 | 0.859 | 0.861 | 0.898 | 0.899 |
| ≥85 years | 0.696 | 0.764 | 0.797 | 0.819 | 0.853 | 0.879 | 0.919 | 0.919 | 0.928 |

**HFRS:** Hospital frailty risk score; **CCI:** Charlson Comorbidity Index

S9c Table. Area Under ROC for 9 periods of prediction long length of stay and 8 age groups for HFRS combined with gender and elective admissions.

| Subset data | **HFRS + gender models** | | | | | | | | |
| --- | --- | --- | --- | --- | --- | --- | --- | --- | --- |
|  | **Length of Stay (LOS) group** | | | | | | | | |
|  | **LOS >3 days** | **LOS >7 days** | **LOS >10 days** | **LOS >14 days** | **LOS >21 days** | **LOS >30 days** | **LOS >45 days** | **LOS >60 days** | **LOS >90 days** |
| 16-24 years | 0.683 | 0.752 | 0.765 | 0.775 | 0.782 | 0.800 | 0.726 | 0.824 | NA |
| 25-34 years | 0.668 | 0.776 | 0.796 | 0.796 | 0.779 | 0.748 | 0.807 | 0.722 | NA |
| 35-44 years | 0.669 | 0.760 | 0.764 | 0.784 | 0.848 | 0.849 | 0.780 | 0.888 | NA |
| 45-54 years | 0.628 | 0.743 | 0.772 | 0.798 | 0.846 | 0.848 | 0.813 | 0.763 | 0.901 |
| 55-64 years | 0.646 | 0.745 | 0.768 | 0.790 | 0.834 | 0.856 | 0.873 | 0.905 | 0.902 |
| 65-74 years | 0.622 | 0.727 | 0.762 | 0.792 | 0.826 | 0.857 | 0.899 | 0.903 | 0.925 |
| 75-84 years | 0.615 | 0.711 | 0.749 | 0.788 | 0.838 | 0.856 | 0.856 | 0.891 | 0.881 |
| ≥85 years | 0.677 | 0.747 | 0.784 | 0.814 | 0.849 | 0.878 | 0.904 | 0.921 | 0.873 |

**HFRS:** Hospital frailty risk score; **CCI:** Charlson Comorbidity Index

S9d Table. Area Under ROC for 9 periods of prediction long length of stay and 8 age groups for HFRS combined with CCI and elective admissions.

| Subset data | **HFRS+CCI models** | | | | | | | | |
| --- | --- | --- | --- | --- | --- | --- | --- | --- | --- |
|  | **Length of Stay (LOS) group** | | | | | | | | |
|  | **LOS >3 days** | **LOS >7 days** | **LOS >10 days** | **LOS >14 days** | **LOS >21 days** | **LOS >30 days** | **LOS >45 days** | **LOS >60 days** | **LOS >90 days** |
| 16-24 years | 0.676 | 0.756 | 0.765 | 0.775 | 0.821 | 0.848 | 0.741 | 0.695 | NA |
| 25-34 years | 0.685 | 0.779 | 0.806 | 0.816 | 0.888 | 0.889 | 0.878 | 0.851 | NA |
| 35-44 years | 0.676 | 0.761 | 0.763 | 0.784 | 0.847 | 0.899 | 0.791 | 0.737 | NA |
| 45-54 years | 0.645 | 0.759 | 0.772 | 0.794 | 0.857 | 0.862 | 0.870 | 0.884 | 0.917 |
| 55-64 years | 0.667 | 0.762 | 0.785 | 0.801 | 0.835 | 0.852 | 0.872 | 0.908 | 0.910 |
| 65-74 years | 0.643 | 0.745 | 0.774 | 0.797 | 0.838 | 0.861 | 0.898 | 0.910 | 0.926 |
| 75-84 years | 0.649 | 0.735 | 0.769 | 0.803 | 0.839 | 0.862 | 0.876 | 0.890 | 0.893 |
| ≥85 years | 0.717 | 0.787 | 0.816 | 0.832 | 0.868 | 0.888 | 0.915 | 0.921 | 0.940 |

**HFRS:** Hospital frailty risk score; **CCI:** Charlson Comorbidity Index
