# Supplementary material for: Using the Hospital Frailty Risk Score to predict length of stay across all adult ages
Source: PLoS One. 2025 Jan 23;20(1):e0317234. doi: 10.1371/journal.pone.0317234 (PMC11756769; doi:10.1371/journal.pone.0317234)
Supplement: S11 Table — Area Under ROC for 9 periods of long length of stay for models HFRS alone or combined with one other variable and models CCI alone or combined with one other variable for all admissions, and index admission. (DOCX) [file pone.0317234.s011.docx]

**S11 Table: (S11a-S11d) Tables.** **Area Under ROC for 9 periods of long length of stay for all patients and index admissions for each patient.**

S11a Table. Area Under ROC for 9 periods of prediction long length of stay and **all patients** **for HFRS models**

| All admissions | **LOS > 3 days** | **LOS > 7 days** | **LOS >10 days** | **LOS> 14 days** | **LOS > 21 days** | **LOS > 30 days** | **LOS > 45 days** | **LOS > 60 days** | **LOS > 90 days** |
| --- | --- | --- | --- | --- | --- | --- | --- | --- | --- |
| HFRS alone | 0.779 | 0.827 | 0.841 | 0.853 | 0.867 | 0.875 | 0.880 | 0.880 | 0.890 |
| HFRS+Age | 0.769 | 0.813 | 0.826 | 0.836 | 0.846 | 0.853 | 0.857 | 0.856 | 0.882 |
| HFRS+Gender | 0.779 | 0.827 | 0.841 | 0.853 | 0.865 | 0.871 | 0.878 | 0.874 | 0.888 |
| HFRS+CCI | 0.792 | 0.835 | 0.847 | 0.857 | 0.865 | 0.874 | 0.877 | 0.877 | 0.889 |

S11b Table. Area Under ROC for 9 periods of prediction long length of stay and **index admissions** **for HFRS models**

| index admissions | **LOS > 3 days** | **LOS > 7 days** | **LOS >10 days** | **LOS> 14 days** | **LOS > 21 days** | **LOS > 30 days** | **LOS > 45 days** | **LOS > 60 days** | **LOS > 90 days** |
| --- | --- | --- | --- | --- | --- | --- | --- | --- | --- |
| HFRS alone | 0.775 | 0.843 | 0.862 | 0.878 | 0.893 | 0.904 | 0.911 | 0.914 | 0.919 |
| HFRS+Age | 0.781 | 0.842 | 0.858 | 0.870 | 0.879 | 0.884 | 0.889 | 0.888 | 0.902 |
| HFRS+Gender | 0.777 | 0.841 | 0.861 | 0.878 | 0.893 | 0.904 | 0.908 | 0.909 | 0.915 |
| HFRS+CCI | 0.787 | 0.853 | 0.869 | 0.884 | 0.897 | 0.905 | 0.911 | 0.914 | 0.919 |

S11c Table. Area Under ROC for 9 periods of prediction long length of stay and **all patients** **for CCI** **models**

| All admissions | **LOS > 3 days** | **LOS > 7 days** | **LOS >10 days** | **LOS> 14 days** | **LOS > 21 days** | **LOS > 30 days** | **LOS > 45 days** | **LOS > 60 days** | **LOS > 90 days** |
| --- | --- | --- | --- | --- | --- | --- | --- | --- | --- |
| CCI alone | 0.628 | 0.637 | 0.636 | 0.631 | 0.626 | 0.613 | 0.598 | 0.593 | 0.556 |
| CCI+Age | 0.703 | 0.743 | 0.756 | 0.762 | 0.762 | 0.755 | 0.741 | 0.720 | 0.693 |
| CCI+Gender | 0.632 | 0.641 | 0.640 | 0.635 | 0.633 | 0.620 | 0.607 | 0.608 | 0.593 |
| CCI+HFRS | 0.792 | 0.835 | 0.847 | 0.857 | 0.865 | 0.874 | 0.877 | 0.877 | 0.889 |

S11d Table. Area Under ROC for 9 periods of prediction long length of stay and **index admissions** **for CCI models**

| index admissions | **LOS > 3 days** | **LOS > 7 days** | **LOS >10 days** | **LOS> 14 days** | **LOS > 21 days** | **LOS > 30 days** | **LOS > 45 days** | **LOS > 60 days** | **LOS > 90 days** |
| --- | --- | --- | --- | --- | --- | --- | --- | --- | --- |
| CCI alone | 0.616 | 0.64 | 0.645 | 0.649 | 0.648 | 0.639 | 0.627 | 0.617 | 0.584 |
| CCI+Age | 0.735 | 0.792 | 0.807 | 0.817 | 0.821 | 0.813 | 0.808 | 0.790 | 0.732 |
| CCI+Gender | 0.616 | 0.643 | 0.649 | 0.653 | 0.650 | 0.640 | 0.630 | 0.620 | 0.595 |
| CCI+HFRS | 0.787 | 0.853 | 0.869 | 0.884 | 0.897 | 0.905 | 0.911 | 0.914 | 0.919 |
