# Supplementary material for: Using the Hospital Frailty Risk Score to predict length of stay across all adult ages
Source: PLoS One. 2025 Jan 23;20(1):e0317234. doi: 10.1371/journal.pone.0317234 (PMC11756769; doi:10.1371/journal.pone.0317234)
Supplement: S12 Table — Area Under ROC for 9 periods of long length of stay and 8 age groups for models HFRS alone or combined with one other variable (age, gender, CCI) for index admission. (DOCX) [file pone.0317234.s012.docx]

**S12 Table: (S12a-S12d) Tables. Area Under ROC for 9 periods of long length of stay and 8 age groups for** **models HFRS alone or combined with one other variable (age, gender, CCI) for index admission.**

S12a Table. Area Under ROC for 9 periods of prediction long length of stay and 8 age groups for HFRS alone and index admission.

| Subset data | **HFRS alone models** | | | | | | | | |
| --- | --- | --- | --- | --- | --- | --- | --- | --- | --- |
|  | **Length of Stay (LOS) group** | | | | | | | | |
|  | **LOS >3 days** | **LOS >7 days** | **LOS >10 days** | **LOS >14 days** | **LOS >21 days** | **LOS >30 days** | **LOS >45 days** | **LOS >60 days** | **LOS >90 days** |
| 16-24 years | 0.623 | 0.695 | 0.720 | 0.766 | 0.831 | 0.812 | 0.845 | 0.879 | 0.965 |
| 25-34 years | 0.677 | 0.746 | 0.769 | 0.811 | 0.845 | 0.853 | 0.899 | 0.920 | 0.918 |
| 35-44 years | 0.696 | 0.749 | 0.778 | 0.806 | 0.809 | 0.844 | 0.851 | 0.848 | 0.959 |
| 45-54 years | 0.693 | 0.772 | 0.795 | 0.808 | 0.843 | 0.854 | 0.895 | 0.922 | 0.937 |
| 55-64 years | 0.723 | 0.798 | 0.819 | 0.846 | 0.875 | 0.901 | 0.923 | 0.916 | 0.944 |
| 65-74 years | 0.732 | 0.802 | 0.825 | 0.844 | 0.865 | 0.886 | 0.896 | 0.899 | 0.901 |
| 75-84 years | 0.754 | 0.801 | 0.816 | 0.83 | 0.844 | 0.857 | 0.860 | 0.874 | 0.892 |
| ≥85 years | 0.748 | 0.757 | 0.753 | 0.754 | 0.756 | 0.765 | 0.767 | 0.770 | 0.790 |

**HFRS:** Hospital frailty risk score; **CCI:** Charlson Comorbidity Index

S12b Table. Area Under ROC for 9 periods of prediction long length of stay and 8 age groups for HFRS combined with age and index admission.

| Subset data | **HFRS+age models** | | | | | | | | |
| --- | --- | --- | --- | --- | --- | --- | --- | --- | --- |
|  | **Length of Stay (LOS) group** | | | | | | | | |
|  | **LOS >3 days** | **LOS >7 days** | **LOS >10 days** | **LOS >14 days** | **LOS >21 days** | **LOS >30 days** | **LOS >45 days** | **LOS >60 days** | **LOS >90 days** |
| 16-24 years | 0.625 | 0.689 | 0.72 | 0.765 | 0.857 | 0.848 | 0.848 | 0.903 | 0.904 |
| 25-34 years | 0.676 | 0.74 | 0.763 | 0.812 | 0.853 | 0.843 | 0.897 | 0.887 | 0.919 |
| 35-44 years | 0.699 | 0.75 | 0.78 | 0.802 | 0.802 | 0.84 | 0.831 | 0.809 | 0.909 |
| 45-54 years | 0.696 | 0.77 | 0.794 | 0.806 | 0.837 | 0.843 | 0.893 | 0.927 | 0.918 |
| 55-64 years | 0.727 | 0.799 | 0.819 | 0.843 | 0.876 | 0.903 | 0.916 | 0.914 | 0.953 |
| 65-74 years | 0.732 | 0.8 | 0.821 | 0.838 | 0.86 | 0.879 | 0.894 | 0.902 | 0.899 |
| 75-84 years | 0.751 | 0.796 | 0.812 | 0.826 | 0.841 | 0.854 | 0.849 | 0.869 | 0.888 |
| ≥85 years | 0.747 | 0.755 | 0.751 | 0.751 | 0.754 | 0.764 | 0.767 | 0.77 | 0.792 |

**HFRS:** Hospital frailty risk score; **CCI:** Charlson Comorbidity Index

S12c Table. Area Under ROC for 9 periods of prediction long length of stay and 8 age groups for HFRS combined with gender and index admission.

| Subset data | **HFRS + gender models** | | | | | | | | |
| --- | --- | --- | --- | --- | --- | --- | --- | --- | --- |
|  | **Length of Stay (LOS) group** | | | | | | | | |
|  | **LOS >3 days** | **LOS >7 days** | **LOS >10 days** | **LOS >14 days** | **LOS >21 days** | **LOS >30 days** | **LOS >45 days** | **LOS >60 days** | **LOS >90 days** |
| 16-24 years | 0.636 | 0.71 | 0.726 | 0.771 | 0.852 | 0.881 | 0.898 | 0.893 | 0.985 |
| 25-34 years | 0.682 | 0.749 | 0.763 | 0.791 | 0.845 | 0.849 | 0.852 | 0.886 | 0.962 |
| 35-44 years | 0.702 | 0.751 | 0.775 | 0.803 | 0.8 | 0.844 | 0.825 | 0.831 | 0.981 |
| 45-54 years | 0.692 | 0.769 | 0.791 | 0.809 | 0.836 | 0.854 | 0.885 | 0.907 | 0.905 |
| 55-64 years | 0.727 | 0.801 | 0.821 | 0.846 | 0.873 | 0.9 | 0.92 | 0.916 | 0.946 |
| 65-74 years | 0.732 | 0.804 | 0.826 | 0.844 | 0.864 | 0.887 | 0.898 | 0.899 | 0.908 |
| 75-84 years | 0.753 | 0.799 | 0.815 | 0.829 | 0.844 | 0.857 | 0.86 | 0.873 | 0.89 |
| ≥85 years | 0.748 | 0.756 | 0.753 | 0.754 | 0.756 | 0.763 | 0.764 | 0.763 | 0.787 |

**HFRS:** Hospital frailty risk score; **CCI:** Charlson Comorbidity Index

S12d Table. Area Under ROC for 9 periods of prediction long length of stay and 8 age groups for HFRS combined with CCI and index admission.

| Subset data | **HFRS+CCI models** | | | | | | | | |
| --- | --- | --- | --- | --- | --- | --- | --- | --- | --- |
|  | **Length of Stay (LOS) group** | | | | | | | | |
|  | **LOS >3 days** | **LOS >7 days** | **LOS >10 days** | **LOS >14 days** | **LOS >21 days** | **LOS >30 days** | **LOS >45 days** | **LOS >60 days** | **LOS >90 days** |
| 16-24 years | 0.628 | 0.694 | 0.723 | 0.763 | 0.827 | 0.808 | 0.82 | 0.85 | 0.923 |
| 25-34 years | 0.68 | 0.746 | 0.77 | 0.816 | 0.856 | 0.859 | 0.895 | 0.919 | 0.923 |
| 35-44 years | 0.699 | 0.753 | 0.777 | 0.806 | 0.807 | 0.841 | 0.851 | 0.85 | 0.967 |
| 45-54 years | 0.698 | 0.776 | 0.798 | 0.813 | 0.844 | 0.854 | 0.89 | 0.92 | 0.936 |
| 55-64 years | 0.739 | 0.813 | 0.831 | 0.855 | 0.88 | 0.903 | 0.928 | 0.917 | 0.942 |
| 65-74 years | 0.746 | 0.812 | 0.832 | 0.85 | 0.869 | 0.888 | 0.898 | 0.903 | 0.906 |
| 75-84 years | 0.765 | 0.808 | 0.821 | 0.834 | 0.846 | 0.857 | 0.859 | 0.873 | 0.893 |
| ≥85 years | 0.756 | 0.762 | 0.757 | 0.756 | 0.757 | 0.765 | 0.766 | 0.77 | 0.782 |

**HFRS:** Hospital frailty risk score; **CCI:** Charlson Comorbidity Index
