# Supplementary material for: Using the Hospital Frailty Risk Score to predict length of stay across all adult ages
Source: PLoS One. 2025 Jan 23;20(1):e0317234. doi: 10.1371/journal.pone.0317234 (PMC11756769; doi:10.1371/journal.pone.0317234)
Supplement: S13 Table — Area Under ROC for 9 periods of long length of stay and 8 age groups for models CCI alone or combined with one other variable (age, gender, HFRS) for index admission. (DOCX) [file pone.0317234.s013.docx]

**S13 (S13a-S13d) Table. Area Under ROC for 9 periods of long length of stay and 8 age groups for** **models CCI alone or combined with one other variable (age, gender, HFRS) for index admission.**

S13a Table. Area Under ROC for 9 periods of prediction long length of stay and 8 age groups for CCI alone and index admission.

| Subset data | **CCI alone models** | | | | | | | | |
| --- | --- | --- | --- | --- | --- | --- | --- | --- | --- |
|  | **Length of Stay (LOS) group** | | | | | | | | |
|  | **LOS >3 days** | **LOS >7 days** | **LOS >10 days** | **LOS >14 days** | **LOS >21 days** | **LOS >30 days** | **LOS >45 days** | **LOS >60 days** | **LOS >90 days** |
| 16-24 years | 0.511 | 0.505 | 0.509 | 0.508 | 0.520 | 0.532 | 0.499 | 0.525 | 0.593 |
| 25-34 years | 0.521 | 0.525 | 0.537 | 0.550 | 0.566 | 0.587 | 0.571 | 0.588 | 0.667 |
| 35-44 years | 0.532 | 0.532 | 0.531 | 0.537 | 0.528 | 0.524 | 0.535 | 0.549 | 0.553 |
| 45-54 years | 0.546 | 0.559 | 0.563 | 0.574 | 0.573 | 0.573 | 0.577 | 0.569 | 0.539 |
| 55-64 years | 0.579 | 0.598 | 0.595 | 0.596 | 0.587 | 0.583 | 0.570 | 0.563 | 0.572 |
| 65-74 years | 0.595 | 0.607 | 0.606 | 0.605 | 0.599 | 0.586 | 0.587 | 0.597 | 0.572 |
| 75-84 years | 0.611 | 0.621 | 0.624 | 0.627 | 0.625 | 0.613 | 0.602 | 0.592 | 0.587 |
| ≥85 years | 0.608 | 0.605 | 0.598 | 0.593 | 0.587 | 0.573 | 0.551 | 0.537 | 0.531 |

**HFRS:** Hospital frailty risk score; **CCI:** Charlson Comorbidity Index

S13b Table. Area Under ROC for 9 periods of prediction long length of stay and 8 age groups for CCI combined with age and index admission.

| Subset data | **CCI+age models** | | | | | | | | |
| --- | --- | --- | --- | --- | --- | --- | --- | --- | --- |
|  | **Length of Stay (LOS) group** | | | | | | | | |
|  | **LOS >3 days** | **LOS >7 days** | **LOS >10 days** | **LOS >14 days** | **LOS >21 days** | **LOS >30 days** | **LOS >45 days** | **LOS >60 days** | **LOS >90 days** |
| 16-24 years | 0.514 | 0.505 | 0.514 | 0.511 | 0.550 | 0.575 | 0.544 | 0.606 | 0.783 |
| 25-34 years | 0.536 | 0.552 | 0.545 | 0.557 | 0.588 | 0.576 | 0.544 | 0.627 | 0.713 |
| 35-44 years | 0.548 | 0.549 | 0.545 | 0.541 | 0.529 | 0.533 | 0.534 | 0.566 | 0.620 |
| 45-54 years | 0.553 | 0.575 | 0.578 | 0.598 | 0.592 | 0.599 | 0.619 | 0.630 | 0.626 |
| 55-64 years | 0.614 | 0.635 | 0.635 | 0.640 | 0.637 | 0.630 | 0.640 | 0.621 | 0.651 |
| 65-74 years | 0.607 | 0.628 | 0.627 | 0.631 | 0.625 | 0.622 | 0.633 | 0.646 | 0.586 |
| 75-84 years | 0.635 | 0.654 | 0.657 | 0.657 | 0.654 | 0.644 | 0.640 | 0.626 | 0.638 |
| ≥85 years | 0.637 | 0.639 | 0.636 | 0.629 | 0.622 | 0.604 | 0.572 | 0.516 | 0.540 |

**HFRS:** Hospital frailty risk score; **CCI:** Charlson Comorbidity Index

S13c Table. Area Under ROC for 9 periods of prediction long length of stay and 8 age groups for CCI combined with gender and index admission.

| Subset data | **CCI + gender models** | | | | | | | | |
| --- | --- | --- | --- | --- | --- | --- | --- | --- | --- |
|  | **Length of Stay (LOS) group** | | | | | | | | |
|  | **LOS >3 days** | **LOS >7 days** | **LOS >10 days** | **LOS >14 days** | **LOS >21 days** | **LOS >30 days** | **LOS >45 days** | **LOS >60 days** | **LOS >90 days** |
| 16-24 years | 0.546 | 0.569 | 0.583 | 0.596 | 0.667 | 0.742 | 0.744 | 0.730 | 0.824 |
| 25-34 years | 0.554 | 0.603 | 0.606 | 0.597 | 0.598 | 0.627 | 0.684 | 0.705 | 0.865 |
| 35-44 years | 0.564 | 0.586 | 0.593 | 0.597 | 0.616 | 0.653 | 0.625 | 0.641 | 0.740 |
| 45-54 years | 0.566 | 0.599 | 0.617 | 0.626 | 0.626 | 0.627 | 0.653 | 0.638 | 0.609 |
| 55-64 years | 0.586 | 0.605 | 0.604 | 0.605 | 0.594 | 0.587 | 0.592 | 0.565 | 0.577 |
| 65-74 years | 0.594 | 0.603 | 0.613 | 0.613 | 0.610 | 0.597 | 0.601 | 0.607 | 0.594 |
| 75-84 years | 0.620 | 0.632 | 0.633 | 0.636 | 0.631 | 0.619 | 0.608 | 0.599 | 0.576 |
| ≥85 years | 0.620 | 0.620 | 0.614 | 0.604 | 0.594 | 0.579 | 0.550 | 0.550 | 0.538 |

**HFRS:** Hospital frailty risk score; **CCI:** Charlson Comorbidity Index

S13d Table. Area Under ROC for 9 periods of prediction long length of stay and 8 age groups for CCI combined with HFRS and index admission.

| Subset data | **CCI+HFRS models** | | | | | | | | |
| --- | --- | --- | --- | --- | --- | --- | --- | --- | --- |
|  | **Length of Stay (LOS) group** | | | | | | | | |
|  | **LOS >3 days** | **LOS >7 days** | **LOS >10 days** | **LOS >14 days** | **LOS >21 days** | **LOS >30 days** | **LOS >45 days** | **LOS >60 days** | **LOS >90 days** |
| 16-24 years | 0.628 | 0.694 | 0.732 | 0.763 | 0.827 | 0.808 | 0.820 | 0.850 | 0.923 |
| 25-34 years | 0.680 | 0.746 | 0.770 | 0.816 | 0.856 | 0.859 | 0.895 | 0.919 | 0.923 |
| 35-44 years | 0.699 | 0.753 | 0.777 | 0.806 | 0.807 | 0.841 | 0.851 | 0.850 | 0.967 |
| 45-54 years | 0.698 | 0.776 | 0.798 | 0.813 | 0.844 | 0.854 | 0.890 | 0.920 | 0.936 |
| 55-64 years | 0.739 | 0.813 | 0.831 | 0.855 | 0.880 | 0.903 | 0.928 | 0.917 | 0.942 |
| 65-74 years | 0.746 | 0.812 | 0.832 | 0.850 | 0.869 | 0.888 | 0.898 | 0.903 | 0.906 |
| 75-84 years | 0.765 | 0.808 | 0.821 | 0.834 | 0.846 | 0.857 | 0.859 | 0.873 | 0.893 |
| ≥85 years | 0.756 | 0.762 | 0.757 | 0.756 | 0.757 | 0.765 | 0.766 | 0.777 | 0.782 |

**HFRS:** Hospital frailty risk score; **CCI:** Charlson Comorbidity Index
