# Supplementary material for: Using the Hospital Frailty Risk Score to predict length of stay across all adult ages
Source: PLoS One. 2025 Jan 23;20(1):e0317234. doi: 10.1371/journal.pone.0317234 (PMC11756769; doi:10.1371/journal.pone.0317234)
Supplement: S14 Table — (DOCX) [file pone.0317234.s014.docx]

S14 Table. Correlation between HFRS, CCI, and age

|  | **HFRS & CCI** | **HFRS & age** | **CCI & age** |
| --- | --- | --- | --- |
|  | **Correlation (P-value)** | **Correlation (P-value)** | **Correlation (P-value)** |
| All Patients | 0.26 (0.000) | 0.29 (0.000) | 0.19 (0.000) |
| (16-24) years | 0.09 (0.000) | 0.06 (0.000) | -0.01 (0.085 |
| (25-34) years | 0.17 (0.000) | 0.02 (0.000) | 0.04 (0.000) |
| (35-44) years | 0.12 (0.000) | 0.03 (0.000) | 0.06 (0.000) |
| (45-54) years | 0.16 (0.000) | 0.02 (0.000) | 0.02 (0.000) |
| (55-64) years | 0.17 (0.000) | 0.06 (0.000) | 0.05 (0.000) |
| (65-74) years | 0.19 (0.000) | 0.06 (0.000) | 0.02 (0.000) |
| (75-84) years | 0.26 (0.000) | 0.12 (0.000) | 0.05 (0.000) |
| ≥ 85 years | 0.26 (0.000) | 0.17 (0.000) | 0.05 (0.000) |

**HFRS:** Hospital frailty risk score; **CCI:** Charlson Comorbidity Index
