# Supplementary material for: Using the Hospital Frailty Risk Score to predict length of stay across all adult ages
Source: PLoS One. 2025 Jan 23;20(1):e0317234. doi: 10.1371/journal.pone.0317234 (PMC11756769; doi:10.1371/journal.pone.0317234)
Supplement: S1 Fig — (a) is HFRS models (b) is CCI models. (DOCX) [file pone.0317234.s015.docx]

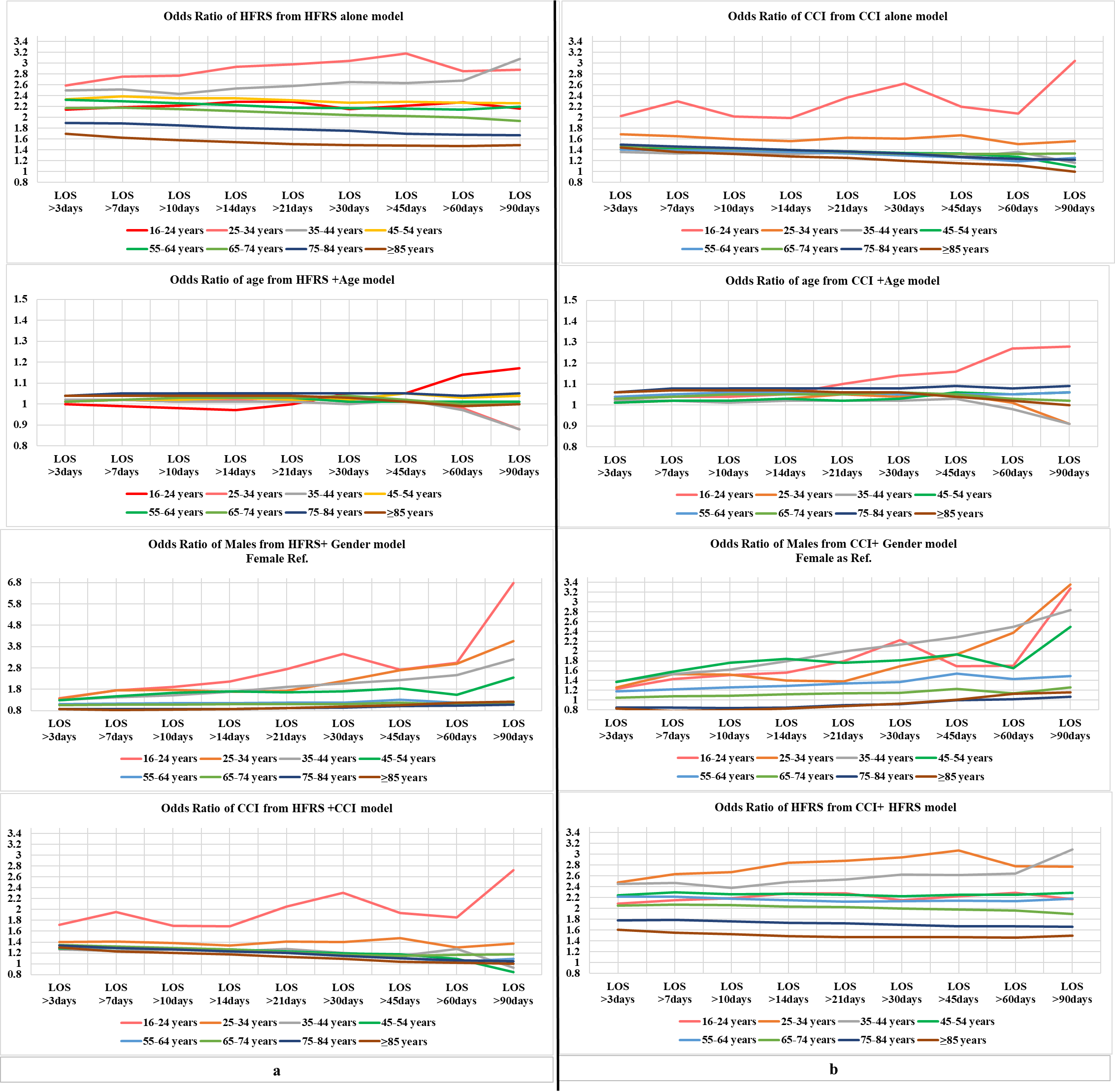


S1 Fig: Logistics regression results (odds ratio) for nine prediction periods of LOS and eight age groups. (a) is HFRS models (b) is CCI models
