# Supplementary material for: Using the Hospital Frailty Risk Score to predict length of stay across all adult ages
Source: PLoS One. 2025 Jan 23;20(1):e0317234. doi: 10.1371/journal.pone.0317234 (PMC11756769; doi:10.1371/journal.pone.0317234)
Supplement: S2 Fig — Area Under ROC for 9 periods of long length of stay and 8 age groups (a) is HFRS models (b) is CCI models. (DOCX) [file pone.0317234.s016.docx]

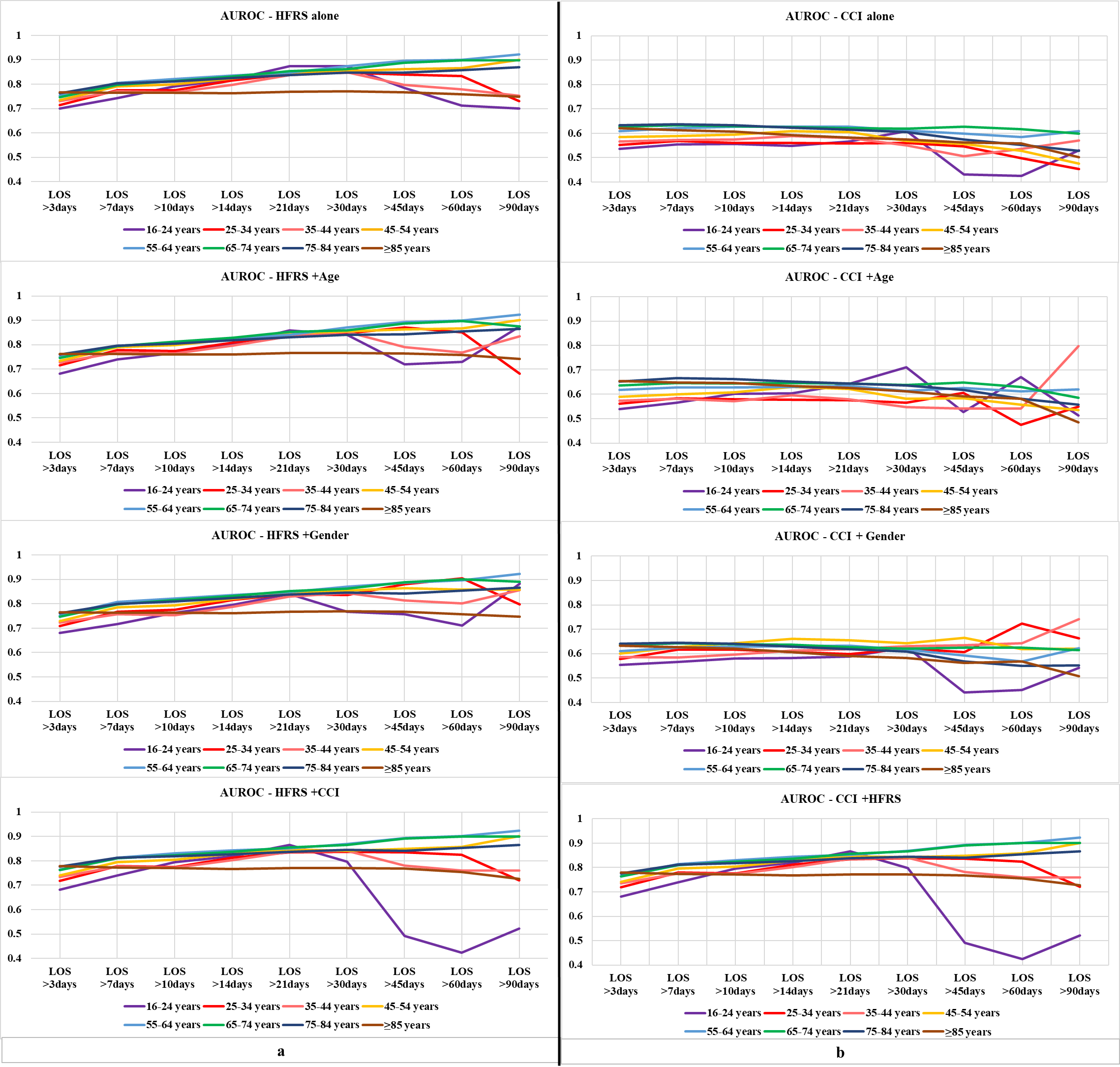


S2 Fig. Area Under ROC for nine periods of prediction long length of stay and eight age groups (a) is HFRS models (b) is CCI models
